# Protective role of ginsenoside Rg1 in the dynamic progression of liver injury to fibrosis: a preclinical meta-analysis

**DOI:** 10.3389/fphar.2025.1512184

**Published:** 2025-01-28

**Authors:** Lijuan Dan, Xiuyan Li, Shuanglan Chen, Xiaojie You, Dong Wang, Tianyuan Wang, Jia Li, Wenping Liu, Jie Mu, Quansheng Feng

**Affiliations:** ^1^ School of Clinical Medicine, Chengdu University of Traditional Chinese Medicine, Chengdu, China; ^2^ School of Basic Medical Sciences, Chengdu University of Traditional Chinese Medicine, Chengdu, China; ^3^ Traditional Chinese Medicine Department, 363 Hospital of Chengdu, Chengdu, China; ^4^ TCM Regulating Metabolic Diseases Key Laboratory of Sichuan Province, Hospital of Chengdu University of Traditional Chinese Medicine, Chengdu, China

**Keywords:** ginsenoside Rg1, liver injury, liver fibrosis, preclinical evidence, meta-analysis

## Abstract

**Background:**

The pathological progression from liver injury to fibrosis is a hallmark of liver disease, with no effective strategies to halt this transition. Ginsenoside Rg1 has demonstrated a range of hepatoprotective properties; however, systematic preclinical evidence supporting its therapeutic potential for liver injury and fibrosis remains limited. Purpose. This study evaluated the efficacy and underlying mechanisms of ginsenoside Rg1 in animal models of liver injury and fibrosis, and providing a basis for future clinical investigation.

**Methods:**

A systematic review was conducted on preclinical studies published in PubMed, Web of Science, and Embase databases up to 1 August 2024, adhereing to rigorous quality standards. The methodological quality was assessed using SYRCLE’s risk of bias tool. Meta-analysis and subgroup analysis were performed using Revman 5.4 software, while publication bias was evaluated through funnel plots and Egger’s test in STATA 15.0 software. Additionally, a time-dose interval curve was utilized to assess the dose-response relationship and identify the effective dose of ginsenoside Rg1 for treating liver injury and fibrosis.

**Results:**

Twenty-four trials involving 423 animals were included. The findings indicated that ginsenoside Rg1 significantly improved liver function markers (ALT and AST), reduced pathological indicators associated with liver injury and fibrosis, and lowered liver fibrosis-related markers (α-SMA, HYP, and PCIII). Furthermore, it exhibited beneficial effects on mechanistic indicators of inflammation, oxidative stress, and apoptosis, compared to the control group (*P* < 0.05). Time-dose interval analysis revealed that the effective dose range of ginsenoside Rg1 was between 4 and 800 mg/kg/d.

**Conclusion:**

Rg1 at a dose of 4–800 mg/kg/d mitigates the progression of liver injury to fibrosis via anti-inflammatory, antioxidative, and anti-apoptotic pathways.

**Systematic Review Registration:**

https://www.crd.york.ac.uk/PROSPERO/, identifier CRD 42024557878.

## Graphical Abstract

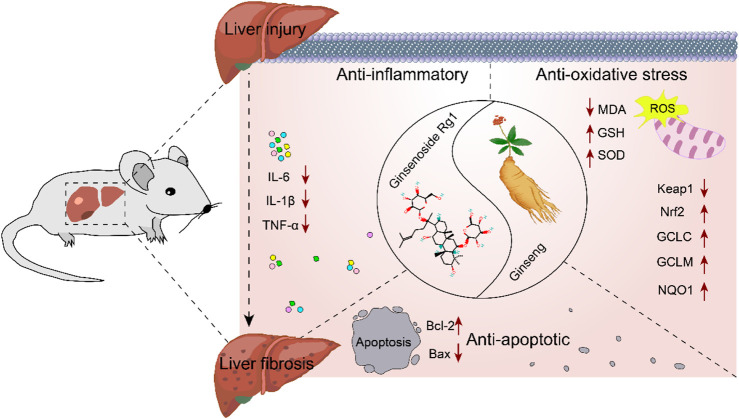

## 1 Introduction

Chronic liver disease (CLD) represents a significant global public health concern, resulting from prolonged liver injury (LI) caused by a range of factors, including infections, trauma, drugs, toxins, and physical and chemical agents (Cools et al., 2024; Guo et al., 2022; Parola and Pinzani, 2019). Although the liver possesses regenerative capabilities, chronic injury often leads to scarring and, if not appropriately managed, may progress to liver fibrosis (LF) (Diehl and Chute, 2013; Ruart et al., 2019). LF is a pathological repair response to persistent LI, characterized by excessive accumulation of extracellular matrix (ECM) proteins and structural degradation of liver tissue (Taru et al., 2024; Zhang et al., 2021). Its incidence is rising globally, and if untreated, it can advance to cirrhosis, hepatocellular carcinoma and liver failure (Zhu et al., 2021) (Yu et al., 2021; Zhang et al., 2022). Hepatic stellate cells (HSCs) play a central role in the development of LF (Wan et al., 2024), with their activation being a critical factor in triggering the fibrotic process (Lu et al., 2021). Inflammation, oxidative stress, and apoptosis are pivotal in driving the dynamic progression from LI to LF (Guicciardi and Gores, 2005; Sharma et al., 2024). These factors activate HSCs, inducing their transformation into myofibroblasts and promoting collagen synthesis, which leads to ECM accumulation and the destruction of hepatic architecture. Prolonged injury, results in ECM replacement of parenchymal cells, forming scar tissue, and exacerbating LF (Bataller and Brenner, 2005; Pydyn et al., 2024; Guan et al., 2016).

**FIGURE 1:**
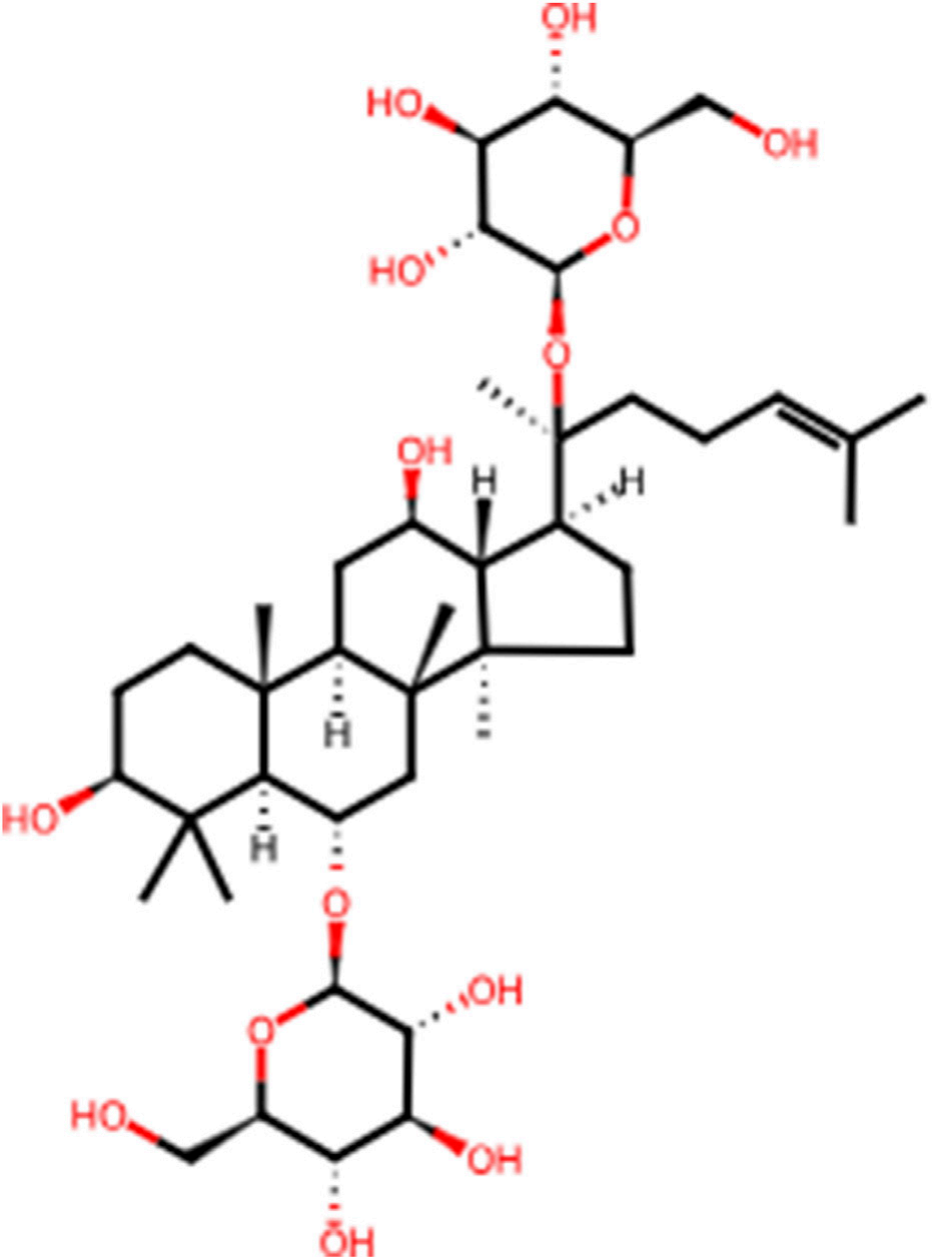
The chemical structure of Rg1.

In recent years, the effectiveness of natural products in halting and reversing the progression of liver disease through various signaling pathways has gained significant attention. *Panax ginseng* C.A.Meyer (ginseng), a perennial herb from the family Wujiaceae and the genus Ginseng, has been used in China for over 2,000 years (Li J. et al., 2022). This traditional and highly valued Chinese herbal medicine is considered the “king of all herbs.” According to The Divine Husbandman’s Classic of the Hundred Herbs, ginseng is classified as a superior product that promotes longevity, replenishes vital energy, and can be consumed over extended periods for health benefits. Ginsenosides, the primary bioactive compounds in ginseng, are chiefly responsible for its pharmacological effects. To date, over 100 ginsenosides have been isolated from Ginseng species, with Rb1, Rb2, Rc, Rd, Rf, and Rg1 accounting for more than 90% of the total ginsenoside content (Alsamman et al., 2018). Among these, Rg1 is one of the most abundant and potent steroidal saponins (Gao et al., 2017a). Ginsenoside Rg1 (C42H72O14, Rg1, Figure 1) plays a key therapeutic role in the progression of LI to LF, including mitigating inflammatory responses and oxidative damage in the early stages and reducing aberrant ECM accumulation following repeated injury (Zhou et al., 2024). Rg1 has also been shown to possess broad therapeutic and prophylactic effects in the central nervous system (Yang et al., 2023), endocrine system (Alolga et al., 2020), and various liver diseases. Its mechanisms of action are believed to involve anti-inflammatory, anti-apoptotic, and antioxidant properties. Despite several preclinical studies highlighting the pharmacological benefits of Rg1 in LI and LF, its comprehensive effects and mechanisms in the dynamic progression from LI to LF remain insufficiently explored. Therefore, the present study aims to investigate the therapeutic effects and underlying mechanisms of Rg1 in the progression from LI to LF, providing essential evidence and preliminary insights for future clinical investigation.

## 2 Materials and methods

This meta-analysis adhere to the PROSPERO protocol (CRD 42024557878) and was conducted in strict compliance with PRISMA guidelines.

### 2.1 Search strategy

A comprehensive search of three databases (PubMed, Embase, and Web of Science) was performed to identify eligible studies investigating the use of Rg1 in LI and LF up to 1 August 2024. The search utilized a combination of subject specific and free text terms. The detailed search strategies for PubMed database are outlined in Supplementary Table S1.

### 2.2 Eligibility criteria

The inclusion criteria were as follows: 1) use of LI or LF as the experimental model; 2) establishment of LI or LF by any method; 3) treatment group receiving any dose of Rg1; 4) if multiple dose groups were included, only the highest dose was selected; 5) primary outcome indicators for LI are: histological score, alanine aminotransferase (ALT) and aminotransferase (AST); secondary outcome indicators for LI are: malondialdehyde (MDA), superoxide dismutase (SOD), glutathione (GSH), kelch-like ECH-associated protein 1 (Keap1), nuclear factor erythroid 2-related factor 2 (Nrf2), glutamate-cysteine ligase modifier subunit (GCLM), glutamate-cysteine ligase catalytic subunit (GCLC), NADH quinone oxidoreductase 1 (NQO1), tumor necrosis factor-α (TNF-α), interleukin 6 (IL-6), interleukin-1β (IL-1β), B-cell lymphoma-2 (Bcl-2), and BCL2-associated X (BAX); 6) primary outcome indicators for LF are: fibrosis score; secondary outcome indicators of LF are hydroxyproline (HYP), α-smooth muscle actin (α-SMA), procollagen type III (PCIII), ALT, and AST.

Exclusion criteria were as follows: 1) non-in vivo studies; 2) non-LI or non-LF models; 3) duplicate publications; 4) non-rodent animal models; 5) studies without Rg1 treatment or lacking a control group; 6) reviews, abstracts, comments, and letters.

### 2.3 Data extraction

Two reviewers (Xiuyan Li and Xiaojie You) independently cconducted the literature search, screened studies, and extracted data based on the inclusion and exclusion criteria, with cross-validation of the data. In cases of disagreement, a third reviewer was involved in the resolution through joint discussion. Data extraction was performed using an Excel sheet that captured the following: 1) basic article details, including the title, first author, and publication year; 2) characteristics of the experimental animals: species (mice or rats), sex (male or female), sample size, weight, and group distribution; 3) modeling methods; 4) intervention details, including drug nature, administration, dosage, and duration; 5) outcome indicators and group differences. The extracted data were compiled and presented in Table 1. For studies with multiple time points, only data from the final time point were included. In cases where different doses of Rg1 were used, only the highest dose was considered. For studies where data were presented graphically, numerical values were extracted using digital ruler software. When data were missing or unclear, the authors were contacted via email for clarification.

**TABLE 1:** Basic characteristics of the 24 included studies.

Study (year)	Disease category	Species (sex, n = treatment/model group, weight)	Modeling method	Intervention (administration, drug, dose, duration)	Outcomes	Intergroup differences
Zhang et al. (2023)	LF	C57BL/6J mice (male, 8/8, N)	10% CCL_4_ (1 mg/kg); intraperitoneal injection; twice a week, 8 weeks	By intragastric, 40 mg/kg/d; 56 days	1.Fibrosis score; 2.α-SMA; 3.HYP	1.*P* < 0.01; 2.*P* < 0.01; 3.*P* < 0.001
Li et al. (2021)	LF	SAMP8 mice (male, 9/9, 30–40 g)	Spontaneous model	By Intragastric, 10 mg/kg/d; 63 days	1.Fibrosis score; 2.PCIII	1.*P* < 0.01; 2.*P* < 0.01
Mo et al. (2021)	LF	C57BL/6 mice (male, 8/8, N)	CCL_4_ (2 mL/kg); intraperitoneal injection; twice a week, 6 weeks	By subcutaneous injection, 40 mg/kg/d; 42 days	1.Fibrosis score; 2.α-SMA	1.*P* < 0.001; 2.*P* < 0.05
Wei et al. (2018)	LF	King-Ming mice (male, 5/5, 23–25 g)	10% CCL_4_ (0.4 mL/kg); subcutaneous injection; twice a week, 6 weeks	By subcutaneous injection, 60 mg/kg/d; 42 days	1.PCIII; 2. HA	1.*P* < 0.05; 2.*P* > 0.05
Li et al. (2014)	LF	Wistar rats (male, 9/8, 200–220 g)	50% CCL_4_ (2 mL/kg); subcutaneous injection; twice a week, 8 weeks	By Intragastric, 40 mg/kg/d; 64 days	1.Fibrosis score; 2.α-SMA; 3. HYP	1.*P* < 0.01; 2.*P* < 0.01; 3.*P* < 0.01
Geng et al. (2010)	LF	Sprague-Dawley rats (N, 10/10, N)	TAA (200 mg/kg); subcutaneous injection; twice a week, 6 weeks	By subcutaneous injection, 100 mg/kg/d; 14 days	1.Fibrosis score; 2. PCIII; 3. HA; 4.HYP	1.*P* < 0.001; 2.*P* < 0.05; 3.*P* < 0.05; 4.*P* < 0.05
Zhou et al. (2024)	LI	C57BL/6J mice (male, 8/8, 17–23 g)	60% CCL_4_ (5 mL/kg); subcutaneous injection; twice a week, 8 weeks	By Intragastric, 800 mg/kg/d; 64 days	1.ALT; 2.AST; 3.IL-1β; 4.IL-6; 5.TNF-α; 6. Keap-1; 7.Bcl-2; 8.BAX	1.*P* < 0.01; 2.*P* < 0.01; 3.*P* < 0.01; 4.*P* < 0.01; 5.*P* < 0.01; 6.*P* < 0.01; 7.*P* < 0.01; 8.*P* < 0.01
Gao et al. (2024)	LI	C57BL/6J mice (male, 6/6, 22–26 g)	ANIT (100 mg/kg); intragastric; 6 days	by Intragastric, 45 mg/kg/d; 6 days	1. ALT; 2.AST; 3.MDA; 4.GSH; 5.SOD; 6.Nrf2; 7.GCLM; 8. GCLC; 9.NQO1	1.*P* < 0.01; 2.*P* < 0.01; 3.*P* < 0.01; 4.*P* < 0.01; 5.*P* < 0.01; 6.*P* < 0.05; 7.*P* < 0.01; 8.*P* < 0.05; 9.*P* < 0.01
Li et al. (2022a)	LI	ICR mice (male, 10/10, 18–22 g)	TCDD (30 μg/kg); subcutaneous injection; per week, 6 weeks	by subcutaneous injection, 200 mg/kg/d; 42 days	1.ALT; 2.AST	1.*P* < 0.001; 2.*P* < 0.001
Jin et al. (2021)	LI	C57BL/6 mice (male, 20/20, 18–20 g)	LPS (100 μg/kg) and D-gal (400 mg/kg); intraperitoneal injection; 3 days	by subcutaneous injection, 30 mg/kg; 3 days	1.ALT; 2.AST; 3.IL-6; 4. TNF-α; 5. MDA; 6. GSH; 7. SOD	1.*P* < 0.001; 2.*P* < 0.001; 3.*P* < 0.001; 4.*P* < 0.001; 5.*P* < 0.001; 6.*P* < 0.001; 7.*P* < 0.001
Zhao et al. (2021)	LI	C57BL/6 mice (male, 3/3, N)	50% CCL_4_ (2 mL/kg); subcutaneous injection; once	By intraperitoneal injection, 4 mg/mL/d; 1 day	1.ALT; 2.AST; 3.IL-1β; 4.IL-6; 5.TNF-α	1.*P* < 0.01; 2.*P* < 0.05; 3.*P* < 0.01; 4.*P* < 0.05; 5.*P* < 0.01
Xiao et al. (2018)	LI	C57BL/6J mice (male,10/10,14–16 g)	D-gal (120 mg/kg); intraperitoneal injection; 6 weeks	By subcutaneous injection; 20 mg/kg/d; 32 days	1.ALT; 2.AST; 3.MDA; 4. GSH; 5.SOD	1.*P* < 0.05; 2.*P* < 0.05; 3.*P* < 0.05; 4.*P* < 0.05; 5.*P* < 0.05
Ning et al. (2018c)	LI	C57BL/6J mice (male,10/10, 20–25 g)	APAP (10 mg/kg); intraperitoneal injection; once	By intragastric, 60 mg/kg/d; seven times with an interval of 12 h for 3 consecutive days	1.ALT; 2.AST; 3.Keap-1; 4.MDA; 5.GSH; 6.SOD; 7.Nrf2; 8.Histological score; 9.GCLM; 10.GCLC; 11.NQO1	1.*P* < 0.05; 2.*P* < 0.05; 3.*P* < 0.05; 4.*P* < 0.05; 5.*P* < 0.05; 6.*P* < 0.05; 7.*P* < 0.05; 8.*P* < 0.05; 9.*P* < 0.05; 10.*P* < 0.05; 11. *P* < 0.05
Ning et al. (2018b)	LI	C57BL/6J mice (male,10/10,N)	D-GalN (700 mg/kg) and LPS (40 μg/kg); intraperitoneal injection; once	By intraperitoneal injection, 60 mg/kg/d; 3 days	1.ALT; 2.AST; 3.MDA; 4.GSH; 5.SOD; 6.Histological score	1.*P* < 0.05; 2.*P* < 0.05; 3.*P* < 0.05; 4.P < 0.05; 5.*P* < 0.05; 6.*P* < 0.05
Ning et al. (2018a)	LI	C57BL/6 mice (male,6/6,N)	CCL_4_ (750 μL/kg); intraperitoneal injection; once	By intragastric, 60 mg/kg/d; 7 days	1.ALT; 2.AST; 3.IL-1β; 4. TNF-α; 5.Keap-1; 6.MDA; 7.GSH; 8.SOD; 9.Nrf2; 10.Histological score; 11.GCLM; 12.GCLC; 13.NQO1	1.*P* < 0.05; 2.*P* < 0.05; 3.*P* < 0.05; 4.*P* < 0.05; 5.*P* < 0.05; 6. *P* < 0.05; 7.*P* < 0.05; 8.*P* < 0.05; 9.*P* < 0.05; 10.*P* < 0.05; 11. *P* < 0.05; 12.*P* < 0.05; 13.*P* < 0.05
Qi et al. (2017)	LI	Kunming mice (male, 12/12, 18–22 g)	CCL_4_ (100 mL/kg); intraperitoneal injection; once	By Intragastric, 40 mg/kg/d; 7 days	1.ALT; 2.AST; 3.IL-6; 4.TNF-α; 5.MDA; 6.SOD	1.*P* < 0.05; 2.*P* < 0.05; 3.*P* < 0.05; 4.*P* < 0.05; 5.*P* < 0.05; 6.*P* < 0.05
Yao X et al. (2016)	LI	Kunming mice (male, 10/10, 18–22 g)	0.3% CCL_4_ (10 mL/kg); intraperitoneal injection; once	By Intragastric, 40 mg/kg/d; 7 days	1.ALT; 2. AST; 3.IL-6; 4.TNF-α; 5.MDA; 6.SOD	1.*P* < 0.01; 2.*P* < 0.01; 3.*P* < 0.01; 4.*P* < 0.01; 5.*P* < 0.01; 6.*P* < 0.01
Zhao et al. (2021)	LI	C57BL/6Jmice (male, 10/10,22–25 g)	Surgical interruption of blood supply to the left lateral and median lobes of the liver	By intraperitoneal injection, 20 mg/kg/d; 7 days	1.ALT; 2.AST; 3.Histological score; 4.BAX; 5.Bcl-2	1.*P* < 0.01; 2.*P* < 0.01; 3.*P* < 0.01; 4.*P* < 0.01; 5.*P* < 0.01
Tao et al. (2014)	LI	C57BL/6Jmice (male,6/6,22–30 g)	Surgical ligation of the portal vein and hepatic artery	By intravenous injection; 20 mg/kg/d; 1 day	1.ALT; 2.AST; 3.Histological score	1.*P* < 0.05; 2.*P* < 0.05; 3.*P* < 0.01
Bi et al. (2021)	LI	Kunming mice (female,6/6,22–25 g)	APAP (250 mg/kg); injection; once	By Intragastric, 30 mg/kg/d; 7 days	1.ALT; 2.AST; 3.IL-1β; 4.IL-6; 5.TNF-α; 6.MDA; 7.GSH; 8.SOD; 9.Histological score; 10.Bcl-2; 11.BAX	1.*P* < 0.05; 2.*P* < 0.05; 3.*P* < 0.01; 4.*P* < 0.01; 5.*P* > 0.05; 6. *P* < 0.05; 7.*P* < 0.05; 8.*P* < 0.05; 9.*P* < 0.01; 10.*P* < 0.05; 11.*P* < 0.05
Gao et al. (2017a)	LI	C57BL/6mice (male,10/10,23–25 g)	Cisplatin (2 mL/kg); intraperitoneal injection; once	By Intragastric, 320 mg/kg/d; 5 days	1.ALT; 2.AST; 3.Keap-1; 4.MDA; 5.GSH; 6.Nrf2; 7. GCLM; 8. GCLC; 9. NQO1	1.*P* > 0.05; 2.*P* > 0.05; 3.*P* > 0.01; 4. *P* > 0.05; 5.*P* > 0.01; 6.*P* < 0.01; 7.*P* < 0.01; 8.*P* > 0.05; 9.*P* > 0.05
Gao et al. (2017b)	LI	C57BL/6mice (male,10/10,23–25 g)	fed ethanol-containing liquid diet	By Intragastric, 40 mg/kg/d; 15 days	1.ALT; 2.AST; 3.Nrf2	1.*P* > 0.05; 2.*P* < 0.05; 3.*P* < 0.05
Lu et al. (2018)	LI	ICR mice (male,8/8,N)	0.3% CCL_4_ (10 mg/kg); intraperitoneal injection; once	By intraperitoneal injection; 30 mg/kg/d; 7 days	1.ALT; 2.AST; 3.IL-6	1.*P* < 0.05; 2.*P* < 0.05; 3.*P* < 0.05
Lin et al. (2020)	LI	Sprague-Dawley rats (male,8/8,250–300 g)	Surgical blockade of blood flow to 70% of the rat liver (left and middle lobes)	By tail vein injection; 20 mg/kg/d; 1 day	1.ALT; 2.AST; 3.Histological score	1.*P* < 0.05; 2.*P* < 0.05; 3.*P* < 0.05

LI, liver injury; LF, liver fibrosis; α-SMA, α-smooth muscle actin; HYP, hydroxyproline; ALT, alanine aminotransferase; AST, aspartate aminotransferase; PCIII, procollagen type III; HA, hyaluronic acid; CCL_4_, carbon tetrachloride; HYP, hydroxyproline; TAA, thioacetamide; IL-1β, interleukin-1β; IL-6, interleukin 6; TNF-α, tumor necrosis factor-α; Keap-1, kelch-like ECH-associated protein 1; Bcl-2, B-cell lymphoma-2; BAX, BCL2-ssociated X; ANIT, α-naphthylisothiocyanate; MDA, malondialdehyde; GSH, glutathione; SOD, superoxide dismutase; Nrf2, nuclear factor erythroid 2-related factor 2; ICR, institute of cancer Research; TCDD, 2,3,7,8-tetrachlorodibenzo-p-dioxin; LPS, lipopolysaccharide; D-gal, d-galactose; APAP, acetaminophen; NQO1, NADH, quinone oxidoreductase.

### 2.4 Risk-of-bias assessment

Risk assessment of the included studies was conducted using the risk assessment tool developed by the Systematic Review Center for Laboratory Animal Experiments (SYRCLE) to evaluate the methodological quality of studies on Rg1 for the treatment of liver injury (LI) and liver fibrosis (LF). The evaluation was based on ten assessment items: (1) sequence generation, (2) baseline characterization, (3) allocation concealment, (4) randomization of animal placement, (5) blinding (animal keepers and investigators), (6) randomization of outcome assessment, (7) blinding (outcome evaluators), (8) reporting of incomplete data, (9) reporting of selective outcomes, and (10) other sources of bias. Each study was independently evaluated by two trained individuals, and disagreements were resolved through discussion with the authors of the article.

### 2.5 Statistical analysis

Meta-analysis of the data from the included studies was performed using R 5.4.1 software. Pooled statistics for outcomes were calculated using standardized mean differences (SMDs) and corresponding 95% confidence intervals (95% *CI*). Heterogeneity was accessed quantitatively using I^2^. If no statistical heterogeneity was observed (I^2^ ≤ 50%), a fixed-effects model was used. In the presence of statistical heterogeneity (I^2^ > 50%), a random-effects model was employed. A subsequent subgroup analysis was performed to identify potential sources of heterogeneity categorized by: publication year (before and after 2019), rodent species (rats and mice), drug dosage (<40 mg and ≥40 mg), modeling methods (toxic, surgical, and nutritional), mode of administration (intragastric and injection), and duration of treatment (<7 days and ≥7 days). Time-dose interval analysis was carried out using Origin 2021 software. *P* < 0.05 was considered statistically significant.

### 2.6 Sensitivity analysis

For results exhibiting high heterogeneity, sensitivity analysis was conducted to assess the robustness of the results and to potentially identify the sources of heterogeneity.

### 2.7 Publication bias

Possible publication bias was evaluated using funnel plots and Egger’s test. Publication bias was visually assessed by using the funnel plot and quantitatively analyzed using Egger’s test.

## 3 Results

### 3.1 Identified and eligible studies

**FIGURE 2:**
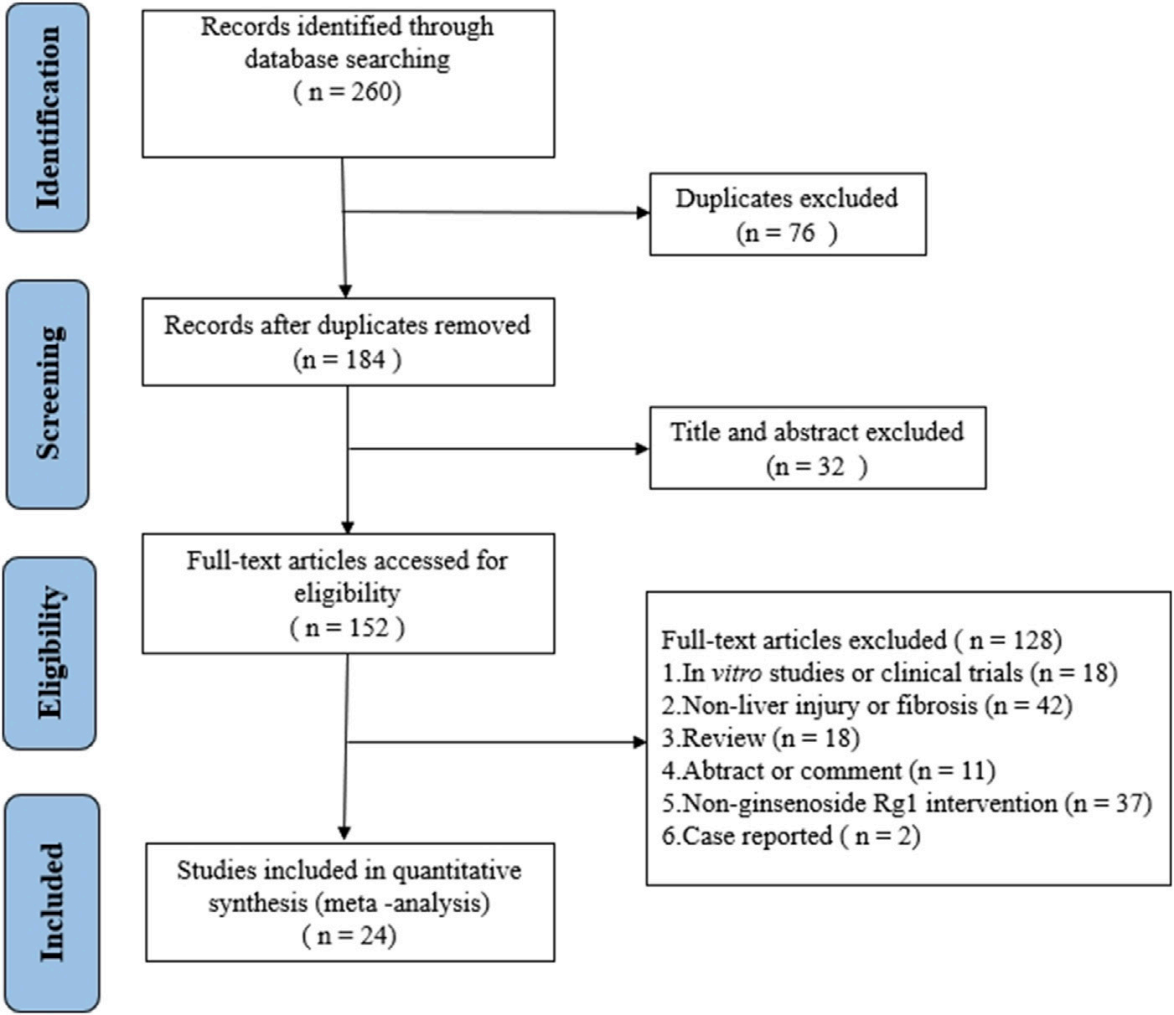
Flow chart of the meta-analysis selection process.

A total of 260 articles were retrieved from three databases via keyword searches: 20 from PubMed, 126 from Web of Science, and 114 from Embase. After automatic weight removal in EndNote, 184 documents remained. Title and abstract screening excluded 32 studies, leaving 152 for further review. Full-text evaluation led to the exclusion of an additional 128 articles, resulting in 24 studies that met the inclusion criteria. The detailed selection process is depicted in Figure 2.

### 3.2 Characteristics of included studies

Twenty-four animal studies (Bi et al., 2021; Gao et al., 2024; Gao et al., 2016; Gao et al., 2017b; Geng et al., 2010; Jin et al., 2021; Li J. et al., 2022; Li et al., 2014; Li et al., 2021; Lin et al., 2020; Lu et al., 2018; Mo et al., 2021; Ning et al., 2018a; Ning et al., 2018b; Ning et al., 2018c; Qi et al., 2017; Tao et al., 2014; Wei et al., 2018; Xiao et al., 2018; Xin et al., 2016; Zhang et al., 2023; Zhang et al., 2015; Zhao et al., 2021; Zhou et al., 2024) conducted between 2010 and 2024 were included. A total of 423 animals from LI and LF models were enrolled, with 212 in the experimental group and 211 in the model group. All studies involved rats or mice, including 14 studies (Gao et al., 2024; Gao et al., 2016; Gao et al., 2017a; Jin et al., 2021; Mo et al., 2021; Ning et al., 2018a; Ning et al., 2018b; Ning et al., 2018c; Tao et al., 2014; Xiao et al., 2018; Zhang et al., 2023; Zhang et al., 2015; Zhao et al., 2021; Zhou et al., 2024) using C57BL/6J mice (250/423,59.1%); 1 study (Li et al., 2021) using SAMP8 mice (18/423, 4.2%); 4 studies (Bi et al., 2021; Qi et al., 2017; Wei et al., 2018; Xin et al., 2016) using Kunming mice (66/423, 15.6%); 1 study (Li et al., 2014) using Wistar rats (17/423, 4%); 2 studies (Geng et al., 2010; Lin et al., 2020) using Sprague-Dawley rats (36/423, 8.5%); 2 studies (Li et al., 2022a; Lu et al., 2018) using ICR mice (36/423, 8.5%). 23 studies (Gao et al., 2017a; Geng et al., 2010; Jin et al., 2021; Li et al., 2022a; Li et al., 2014; Li et al., 2021; Lin et al., 2020; Lu et al., 2018; Mo et al., 2021; Ning et al., 2018a; Ning et al., 2018b; Ning et al., 2018c; Qi et al., 2017; Tao et al., 2014; Wei et al., 2018; Xiao et al., 2018; Xin et al., 2016; Zhang et al., 2023; Zhang et al., 2015; Zhao et al., 2021; Zhou et al., 2024) used male animals, while 1 study (Bi et al., 2021) utilized female animals. 17 studies (Bi et al., 2021; Gao et al., 2024; Gao et al., 2016; Gao et al., 2017b; Jin et al., 2021; Li et al., 2022a; Li et al., 2014; Li et al., 2021; Lin et al., 2020; Ning et al., 2018a; Qi et al., 2017; Tao et al., 2014; Wei et al., 2018; Xiao et al., 2018; Xin et al., 2016; Zhang et al., 2015; Zhou et al., 2024) provided animal weight data. In constructing LI and LF models, 19 studies (Mo et al., 2021; Wei et al., 2018; Zhang et al., 2023) (Bi et al., 2021; Gao et al., 2024; Gao et al., 2017a; Geng et al., 2010; Jin et al., 2021; Li et al., 2022a; Li et al., 2014; Lu et al., 2018; Ning et al., 2018a; Ning et al., 2018b; Ning et al., 2018a; Qi et al., 2017; Xiao et al., 2018; Xin et al., 2016; Zhao et al., 2021; Zhou et al., 2024) used toxic agents, including carbon tetrachloride (CCL_4_), thioacetamide (TAA), α-naphthylisothiocyanate (ANIT), 2,3,7,8-tetrachlorodibenzo-p-dioxin (TCDD), d-galactose (D-gal), cisplatin, and acetaminophen (APAP), three studies (Lin et al., 2020; Tao et al., 2014; Zhang et al., 2015) employed surgical methods, 1 study (Li et al., 2021) used genetic induction, and 1 study (Gao et al., 2016) utilized nutritional factors. The dosing duration ranged from 1 day to 64 days, with Rg1 dose ranging from 4 mg/kg/d to 800 mg/kg/d. For primary outcome indicators of LI and LF, 18 studies (Zhou et al., 2024) (Gao et al., 2024; Li J. et al., 2022) (Bi et al., 2021; Gao et al., 2016; Gao et al., 2017a; Jin et al., 2021; Lin et al., 2020; Lu et al., 2018; Ning et al., 2018a; Ning et al., 2018b; Ning et al., 2018c; Qi et al., 2017; Tao et al., 2014; Xiao et al., 2018; Xin et al., 2016; Zhang et al., 2015; Zhao et al., 2021) reported ALT levels, 18 studies (Zhou et al., 2024) (Gao et al., 2024; Li et al., 2022a) (Bi et al., 2021; Gao et al., 2016; Gao et al., 2017a; Jin et al., 2021; Lin et al., 2020; Lu et al., 2018; Ning et al., 2018a; Ning et al., 2018b; Ning et al., 2018c; Qi et al., 2017; Tao et al., 2014; Xiao et al., 2018; Xin et al., 2016; Zhang et al., 2015; Zhao et al., 2021) reported AST levels, 7 studies (Bi et al., 2021; Lin et al., 2020; Ning et al., 2018b; Ning et al., 2018c; Tao et al., 2014; Zhang et al., 2015; Ning et al., 2018a) documented histological score, and 5 studies (Geng et al., 2010; Li et al., 2014; Li et al., 2021; Mo et al., 2021; Zhang et al., 2023) included fibrosis score. Several studies also reported fibrosis-related markers such as PCIII, HYP, and α-SMA. Inflammatory markers, including IL-6 (Bi et al., 2021; Jin et al., 2021; Lu et al., 2018; Qi et al., 2017; Xin et al., 2016; Zhao et al., 2021; Zhou et al., 2024), IL-1β (Bi et al., 2021; Ning et al., 2018b; Zhao et al., 2021; Zhou et al., 2024), and TNF-α (Bi et al., 2021; Jin et al., 2021; Ning et al., 2018b; Qi et al., 2017; Xin et al., 2016; Zhao et al., 2021; Zhou et al., 2024) were noted in some studies. Additionally, oxidative stress related indicators including MDA (Bi et al., 2021; Gao et al., 2024; Gao et al., 2017a; Jin et al., 2021; Ning et al., 2018a; Ning et al., 2018b; Ning et al., 2018c; Qi et al., 2017; Xiao et al., 2018; Xin et al., 2016), SOD (Bi et al., 2021; Gao et al., 2024; Jin et al., 2021; Ning et al., 2018a; Ning et al., 2018b; Ning et al., 2018c; Qi et al., 2017; Xiao et al., 2018; Xin et al., 2016), and GSH (Bi et al., 2021; Gao et al., 2024; Gao et al., 2017b; Jin et al., 2021; Ning et al., 2018a; Ning et al., 2018b; Ning et al., 2018c; Xiao et al., 2018), were also reported in several studies. Additionally, oxidative stress mechanisms related indicators, including Keap1 (Gao et al., 2017b; Ning et al., 2018a; Ning et al., 2018b; Zhou et al., 2024), Nrf2 (Gao et al., 2024; Gao et al., 2016; Gao et al., 2017a; Ning et al., 2018a; Ning et al., 2018b), GCLC (Q. Gao et al., 2024; Gao et al., 2017a; Ning et al., 2018a; Ning et al., 2018b), GCLM (Gao et al., 2024; Gao et al., 2017a; Ning et al., 2018a; Ning et al., 2018b), and NQO1 (Gao et al., 2024; Gao et al., 2016; Ning et al., 2018a; Ning et al., 2018b) were documented. Apoptosis markers such as Bcl-2 (Bi et al., 2021; Zhang et al., 2015; Zhou et al., 2024) and BAX (Bi et al., 2021; Zhang et al., 2015; Zhou et al., 2024) were included in some studies. Detailed study characteristics are summarized in Table 1.

### 3.3 Research quality

The 24 included articles were rigorously accessed for quality, with all studies scoring moderately or higher. One study (Zhang et al., 2023) received a score of 5, seven studies (Gao et al., 2024; Gao et al., 2017a; Geng et al., 2010; Mo et al., 2021; Ning et al., 2018b; Zhang et al., 2015; Zhao et al., 2021) scored 6, and sixteen studies (Bi et al., 2021; Gao et al., 2016; Jin et al., 2021; Li et al., 2022a; Li et al., 2014; Li et al., 2021; Lin et al., 2020; Lu et al., 2018; Ning et al., 2018a; Ning et al., 2018b; Qi et al., 2017; Tao et al., 2014; Wei et al., 2018; Xiao et al., 2018; Xin et al., 2016; Zhou et al., 2024) scored 7, for a mean quality score of 66.25%. Notably, seven studies (Gao et al., 2024; Gao et al., 2017b; Geng et al., 2010; Ning et al., 2018b; Zhang et al., 2023; Zhang et al., 2015; Zhao et al., 2021) did not report randomization, and two studies (Mo et al., 2021; Zhang et al., 2023) failed to datail the housing conditions of the experimental animals. All studies reported baseline characteristics and conducted randomized outcome analyses, with no instance of incomplete or selectively reported data, and no other sources of bias were identified. However, certain limitation were noted, including the absence of details on allocation concealment, blinding of animal caretakers and investigators, and blinding of outcome assessors. Overall, after quality assessment, the literature was deemed suitable for meta-analysis (Supplementary Table S2).

### 3.4 Effects of Rg1 on LI

#### 3.4.1 Primary outcomes

##### 3.4.1.1 Effect of Rg1 on LI histological score

**FIGURE 3:**
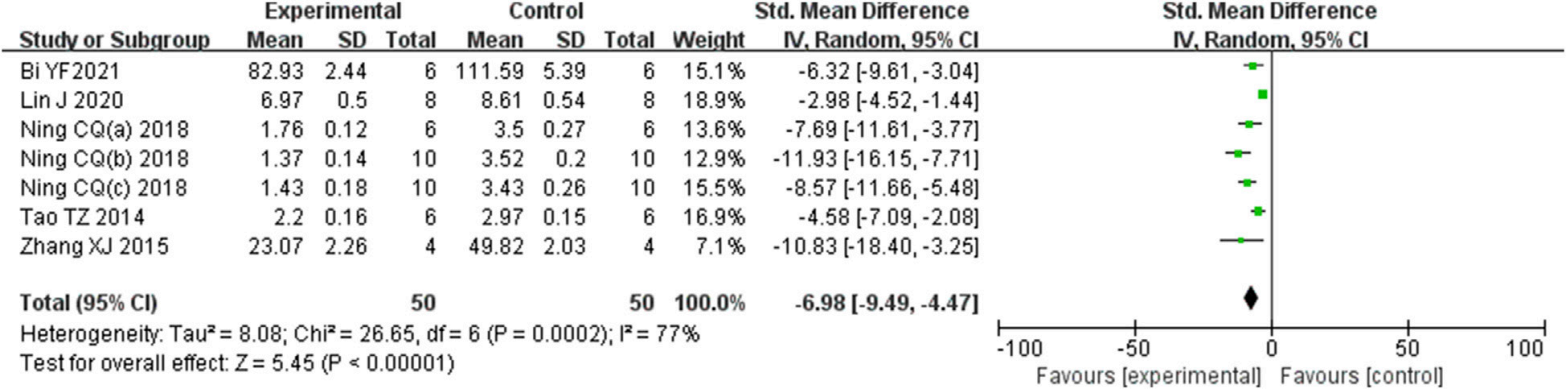
Forest plot: effect of Rg1 on histological score.

Analysis of seven studies (Bi et al., 2021; Lin et al., 2020; Ning et al., 2018b; Ning et al., 2018c; Tao et al., 2014; Zhang et al., 2015; Ning et al., 2018b) involving 100 animals, which reported histological scores, demonstrated that the Rg1 group significantly reduced histological scores compared to the control group [SMD: −6.98 (95% *CI*: −9.49, −4.47), *P* < 0.00001, I^2^ = 77%, Figure 3].

##### 3.4.1.2 Effect of Rg1 on LI ALT level

**FIGURE 4:**
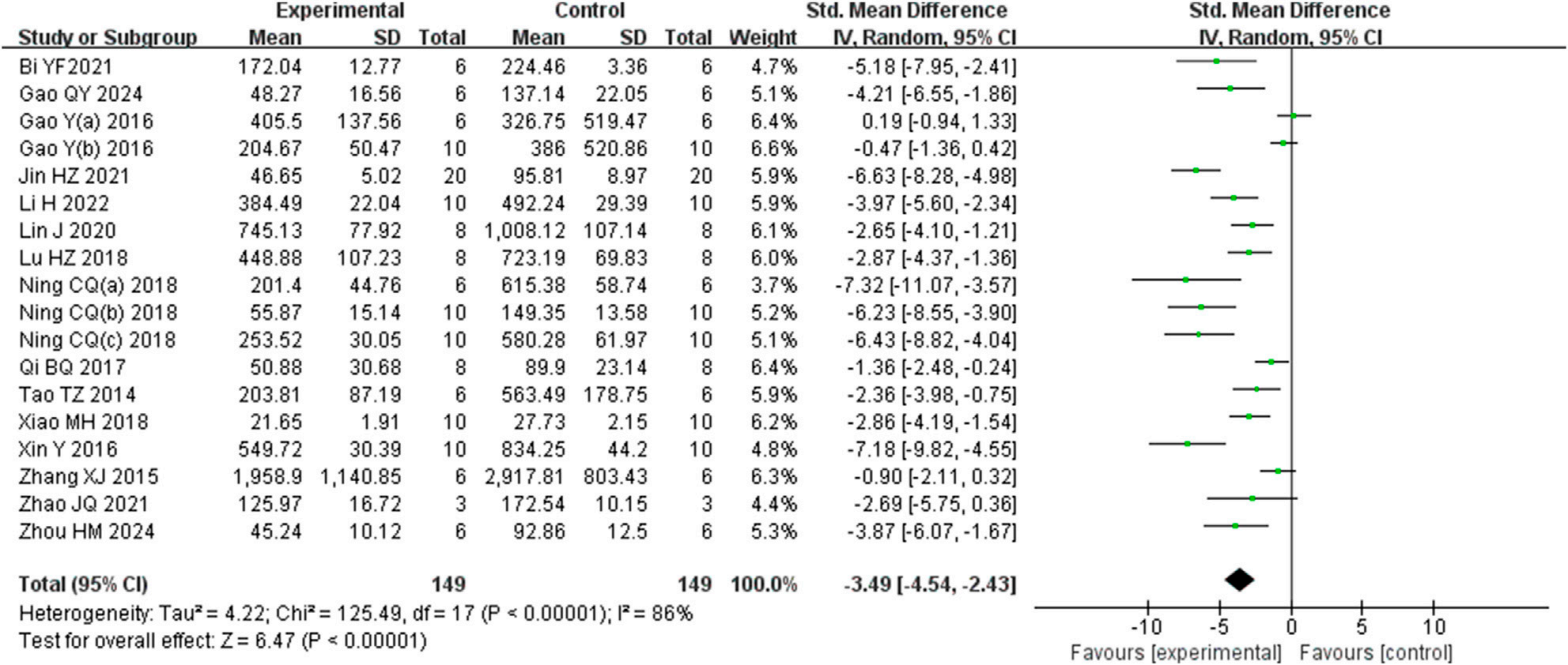
Forest plot: effect of Rg1 on ALT.

Analysis of eighteen studies (Zhou et al., 2024) (Gao et al., 2024; Li J. et al., 2022) (Bi et al., 2021; Gao et al., 2016; Gao et al., 2017a; Jin et al., 2021; Lin et al., 2020; Lu et al., 2018; Ning et al., 2018a; Ning et al., 2018b; Ning et al., 2018c; Qi et al., 2017; Tao et al., 2014; Xiao et al., 2018; Xin et al., 2016; Zhang et al., 2015; Zhao et al., 2021) involving 298 animals, reporting ALT levels, indicated that the Rg1 group significantly reduced ALT compared to the control group [SMD: −3.49 (95% *CI*: −4.54, −2.43), *P* < 0.00001, I^2^ = 86%, Figure 4].

##### 3.4.1.3 Effect of Rg1 on LI AST level

**FIGURE 5:**
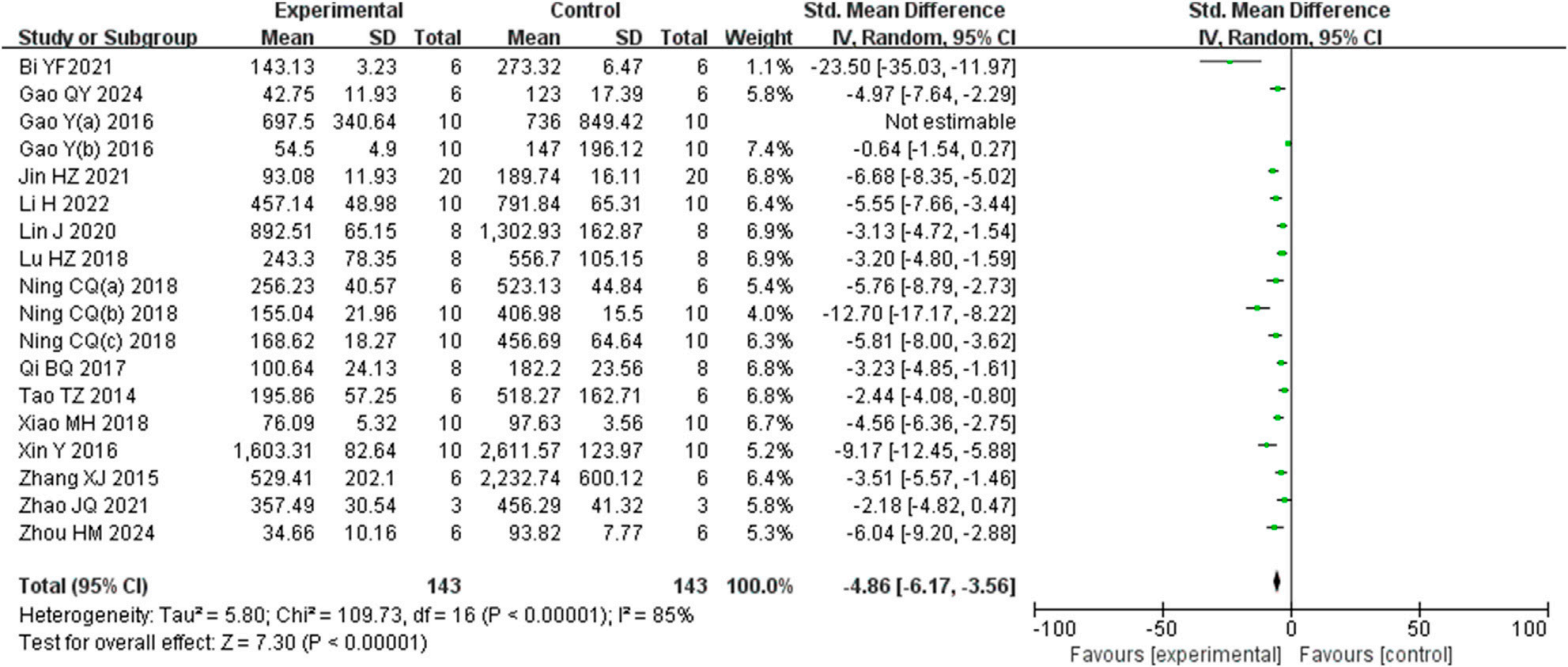
Forest plot: effect of Rg1 on AST.

Analysis of eighteen studies (Zhou et al., 2024) (Gao et al., 2024; Li et al., 2022a) (Bi et al., 2021; Gao et al., 2016; Gao et al., 2017b; Jin et al., 2021; Lin et al., 2020; Lu et al., 2018; Ning et al., 2018a; Ning et al., 2018b; Ning et al., 2018c; Qi et al., 2017; Tao et al., 2014; Xiao et al., 2018; Xin et al., 2016; Zhang et al., 2015; Zhao et al., 2021) involving 286 animals reporting AST levels, indicted that the Rg1 group significantly reduced AST compared to the control group (SMD: −4.86 [95% *CI*: −6.17, −3.56], *P* < 0.00001, I^2^ = 85%, Figure 5).

#### 3.4.2 Secondary outcomes

##### 3.4.2.1 Inflammation levels

**FIGURE 6:**
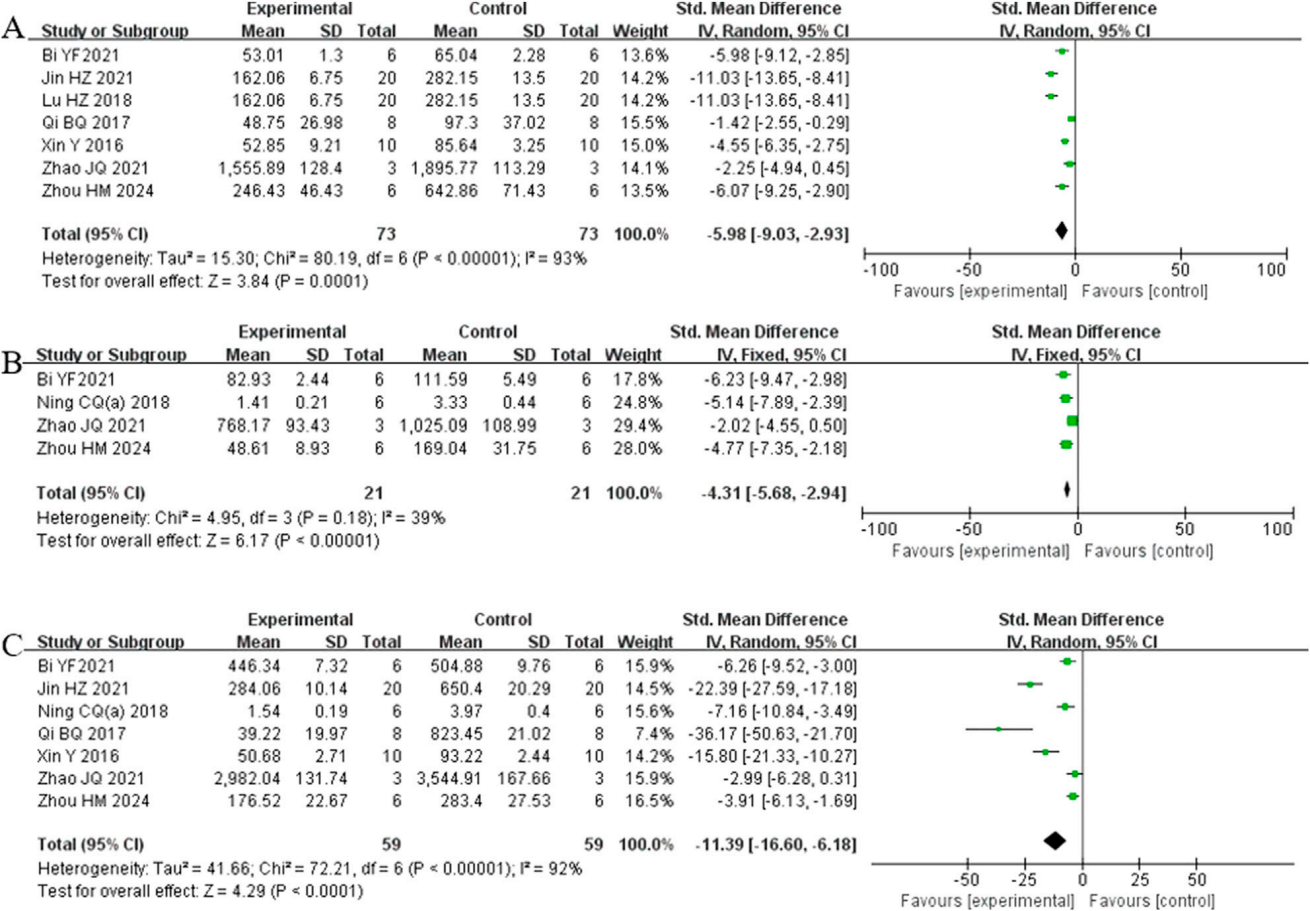
Forest plot: effect of Rg1 on **(A)** IL-6, **(B)** IL-1β, and **(C)** TNF-α.

Analysis of seven studies (Bi et al., 2021; Jin et al., 2021; Lu et al., 2018; Qi et al., 2017; Xin et al., 2016; Zhao et al., 2021; Zhou et al., 2024) involving 146 animals reporting IL-6 levels revealed that the Rg1 group significantly reduced IL-6 compared to the control group [SMD: −5.98 (95% *CI*: −9.03, −2.93), *P* = 0.0001, I^2^ = 93%, Figure 6A]. Analysis of four studies (Bi et al., 2021; Ning et al., 2018b; Zhao et al., 2021; Zhou et al., 2024) involving 42 animals reporting IL-1β levels showed that the Rg1 group significantly reduced IL-1β compared to the control group [SMD: −4.31 (95% *CI*: −5.68, −2.94), *P* < 0.00001, I^2^ = 39%, Figure 6B]. Analysis of seven studies (Bi et al., 2021; Jin et al., 2021; Ning et al., 2018b; Qi et al., 2017; Xin et al., 2016; Zhao et al., 2021; Zhou et al., 2024) involving 118 animals reporting TNF-α levels indicated that the Rg1 group significantly reduced TNF-α compared to the control group [SMD: −11.39 (95% *CI*: −16.60, −6.18), *P* < 0.0001, I^2^ = 92%, Figure 6C].

##### 3.4.2.2 Oxidative stress index

**FIGURE 7:**
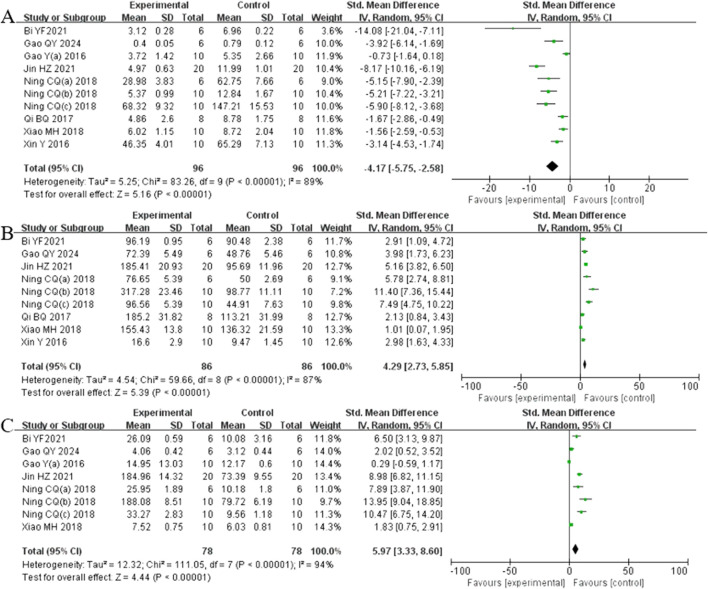
Forest plot: effect of Rg1 on **(A)** MDA, **(B)** SOD, and **(C)** GSH.

Analysis of ten studies (Bi et al., 2021; Gao et al., 2024; Gao et al., 2017b; Jin et al., 2021; Ning et al., 2018a; Ning et al., 2018b; Ning et al., 2018c; Qi et al., 2017; Xiao et al., 2018; Xin et al., 2016) involving 192 animals reporting MDA levels demonstrated that the Rg1 group significantly reduced MDA compare to the control group [SMD: −4.17 (95% *CI*: −5.75, −2.58), *P* < 0.00001, I^2^ = 89%, Figure 7A]. Analysis of nine studies (Y. Bi et al., 2021; Gao et al., 2024; Jin et al., 2021; Ning et al., 2018a; Ning et al., 2018b; Ning et al., 2018c; Qi et al., 2017; Xiao et al., 2018; Xin et al., 2016) involving 192 animals reporting SOD levels revealed that the Rg1 group significantly increased SOD compared to the control group [SMD: 4.29 (95% *CI*: 2.73, 5.85), *P* < 0.00001, I^2^ = 87%, Figure 7B]. Analysis of eight studies (Bi et al., 2021; Gao et al., 2024; Gao et al., 2017a; Jin et al., 2021; Ning et al., 2018a; Ning et al., 2018b; Ning et al., 2018c; Xiao et al., 2018) involving 156 animals reporting GSH levels showed that Rg1 group significantly increased GSH compared to the control group [SMD: 5.97 (95% *CI*: 3.33, 8.60), *P* < 0.00001, I^2^ = 94%, Figure 7C].

##### 3.4.2.3 Indicators related to oxidative stress mechanisms

**FIGURE 8:**
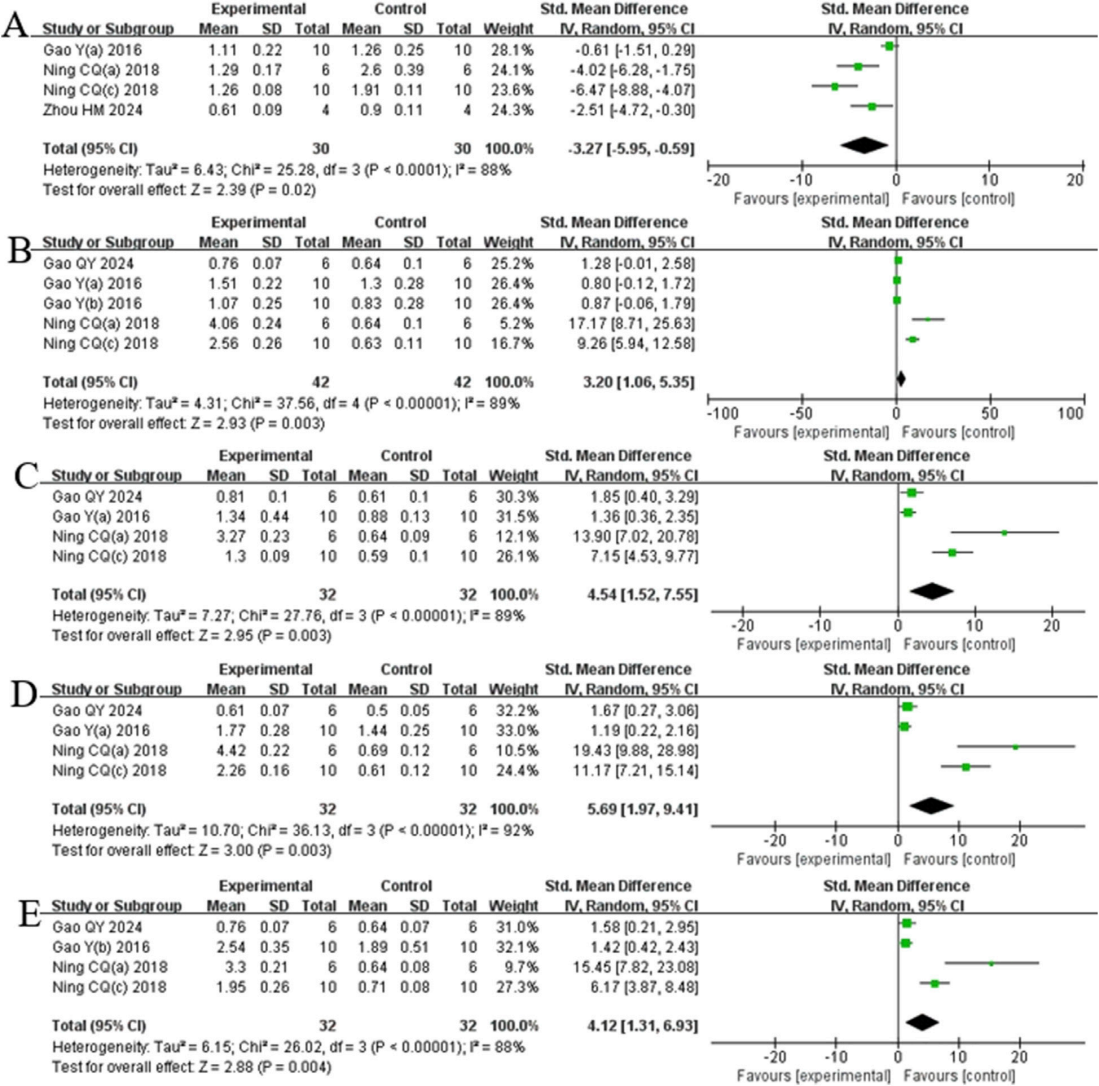
Forest plot: effect of Rg1 on **(A)** Keap1, **(B)** Nrf2, **(C)** GCLM, **(D)** GCLC, and **(E)** NQO1.

Analysis of four studies (Gao et al., 2017a; Ning et al., 2018a; Ning et al., 2018b; Zhou et al., 2024) involving 60 animals reporting Keap1 levels demonstrated that the Rg1 group significantly reduced Keap1 compared to the control group [SMD: −3.27 (95% *CI*: −5.95, −0.59), *P* = 0.02, I^2^ = 88%, Figure 8A]. Analysis of five studies (Gao et al., 2024; Gao et al., 2016; Gao et al., 2017a; Ning et al., 2018a; Ning et al., 2018b) involving 84 animals reporting Nrf2 levels showed that the Rg1 group significantly increased Nrf2 compared to the control group [SMD: 3.20 (95% *CI*: 1.06, 5.35), *P* = 0.003, I^2^ = 89%, Figure 8B]. Analysis of four studies (Gao et al., 2024; Gao et al., 2017b; Ning et al., 2018a; Ning et al., 2018b) involving 64 animals reporting GCLM levels revealed that the Rg1 group significantly increased GCLM compared to the control group [SMD: 4.54 (95% *CI*: 1.52, 7.55), *P* = 0.003, I^2^ = 89%, Figure 8C]. Analysis of four studies (Q. Gao et al., 2024; Gao et al., 2017a; Ning et al., 2018a; Ning et al., 2018b) involving 64 animals reporting GCLC levels indicated that the Rg1 group significantly increased GCLC compared to the control group [SMD: 5.69 (95% *CI*: 1.97, 9.41), *P* = 0.003, I^2^ = 92%, Figure 8D]. Analysis of four studies (Q. Gao et al., 2024; Gao et al., 2016; Ning et al., 2018a; Ning et al., 2018b) involving 64 animals reporting NQO1 levels showed that the Rg1 group significantly increased NQO1 compared to the control group [SMD: 4.12 (95% *CI*: 1.31, 6.93), *P* = 0.004, I^2^ = 88%, Figure 8E].

##### 3.4.2.4 Apoptosis index

**FIGURE 9:**
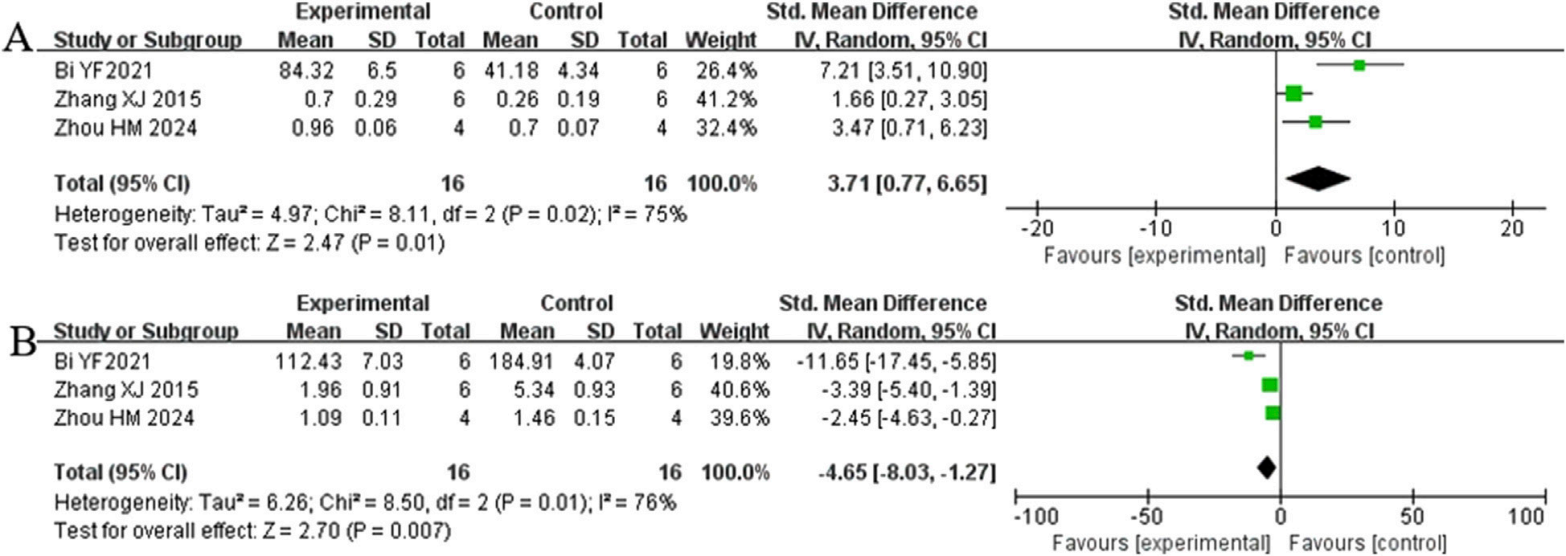
Forest plot: effect of Rg1 on **(A)** Bcl-2 and **(B)** BAX.

Analysis of three studies (Bi et al., 2021; Zhang et al., 2015; Zhou et al., 2024) involving 32 animals reporting Bcl-2 levels revealed that the Rg1 group significantly increased Bcl-2 compared to the control group [SMD: 3.71 (95% *CI*: 0.77, 6.65), *P* = 0.01, I^2^ = 75%, Figure 9A]. Analysis of three studies (Bi et al., 2021; Zhang et al., 2015; Zhou et al., 2024) involving 32 animals reporting BAX levels indicated that Rg1 group significantly decreased BAX compared to the control group [SMD: −4.65 (95% *CI*: −8.03, −1.27), *P* = 0.007, I^2^ = 76%, Figure 9B].

### 3.5 Effects of Rg1 on LF

#### 3.5.1 Primary outcomes

##### 3.5.1.1 Effect of Rg1 on liver fibrosis score in LF

**FIGURE 10:**
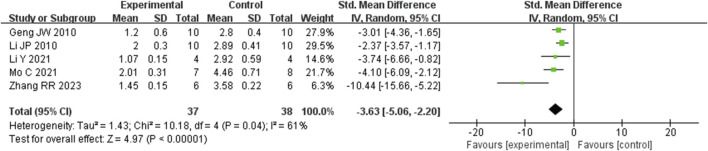
Forest plot: effect of Rg1 on fibrosis score.

Analysis of five studies (Geng et al., 2010; Li et al., 2014; Li et al., 2021; Mo et al., 2021; Zhang et al., 2023) involving 75 animals reporting the fibrosis score levels revealed that the Rg1 group significantly decreased the fibrosis score compared to the control group [SMD: −3.63 (95% *CI*: −5.06, - 2.20), *P* < 0.00001, I^2^ = 61%, Figure 10].

#### 3.5.2 Secondary outcomes

##### 3.5.2.1 Liver fibrosis related indicators

**FIGURE 11:**
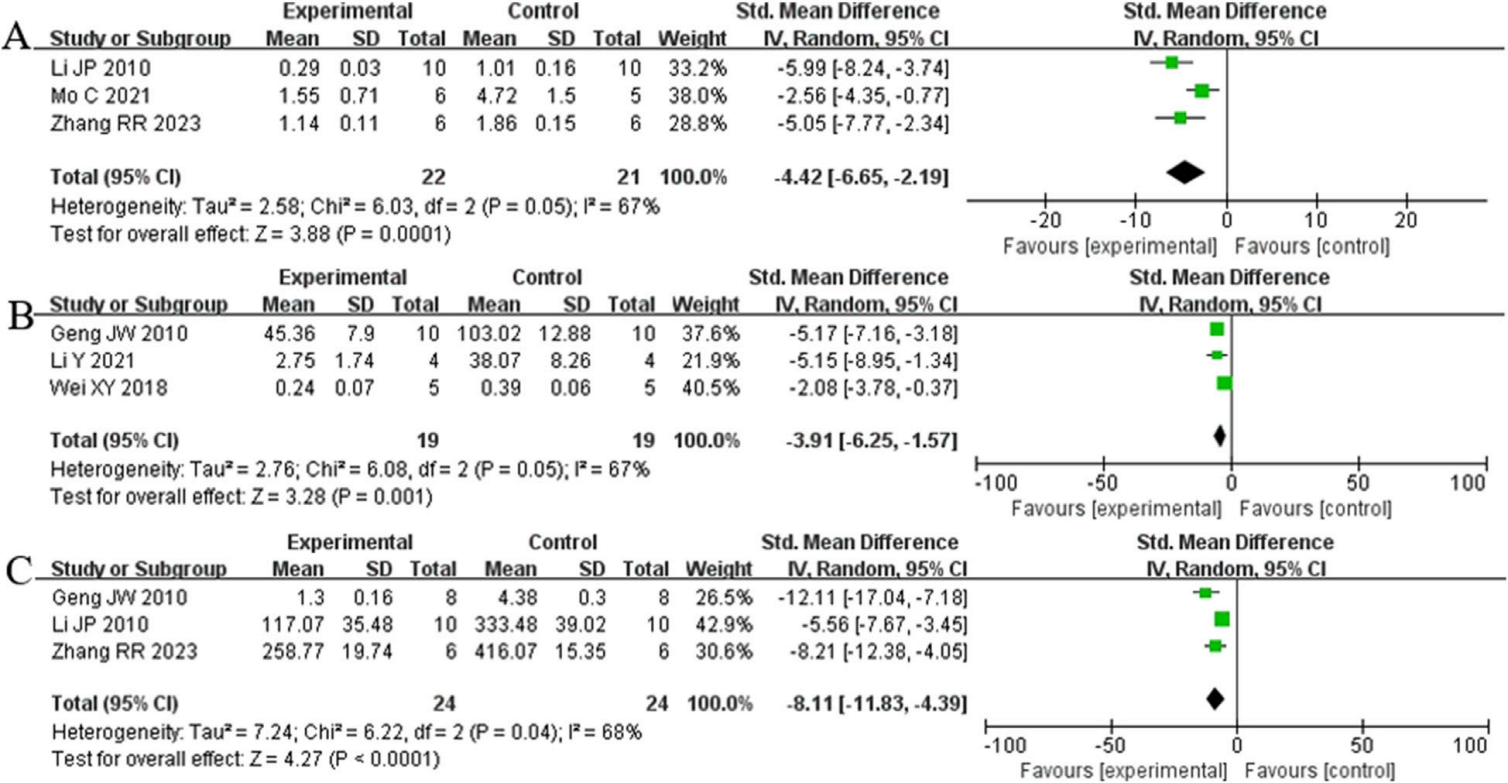
Forest plot: effect of Rg1 on **(A)** α-SMA, **(B)** PCIII, and **(C)** HYP.

Analysis of three studies (Zhang et al., 2023) (Li et al., 2014; Mo et al., 2021) involving 43 animals reporting α-SMA levels showed that the Rg1 group significantly reduced α-SMA compared to the control group [SMD: −4.42 (95% *CI*: −6.65, - 2.19), *P* = 0.0001, I^2^ = 67%, Figure 11A]. Analysis of three studies (Geng et al., 2010; Li et al., 2021; Wei et al., 2018) involving 38 animals reporting PCIII levels indicated that the Rg1 group significantly reduced PCIII compared to the control group [SMD: - 3.91 (95% *CI*: −6.25, - 1.57), *P* = 0.001, I^2^ = 67%, Figure 11B]. Analysis of three studies (Geng et al., 2010; Li et al., 2014; Zhang et al., 2023) involving 48 animals reporting HYP levels demonstrated that the Rg1 group significantly reduced HYP compared to the control group [SMD: - 8.11 (95% *CI*: −11.83, - 4.39), *P* < 0.0001, I^2^ = 68%, Figure 11C].

### 3.6 Subgroup analysis

Given the significant heterogeneity among the included studies, subgroup analysis was performed for histological score, ALT, AST, and fibrosis score based on various factors such as the year of publication, animal species, dosage, modeling method, treatment duration, and administration method. The analysis suggested that modeling method could be the source of heterogeneity for ALT and AST; dosage, modeling method, duration of treatment, and administration method may contribute to the heterogeneity in histological score. Year of publication and dosage were found to be potential source of heterogeneity in fibrosis score. The results are summarized in Supplementary Table S3.

### 3.7 Sensitivity analysis

Additionally, a sensitivity analysis was conducted using the exclusion-by-exclusion method. Sequential exclusion of each study revealed no significant change in the combined results, indicating the robustness and high stability of the findings in this study.

### 3.8 Publication bias

**FIGURE 12:**
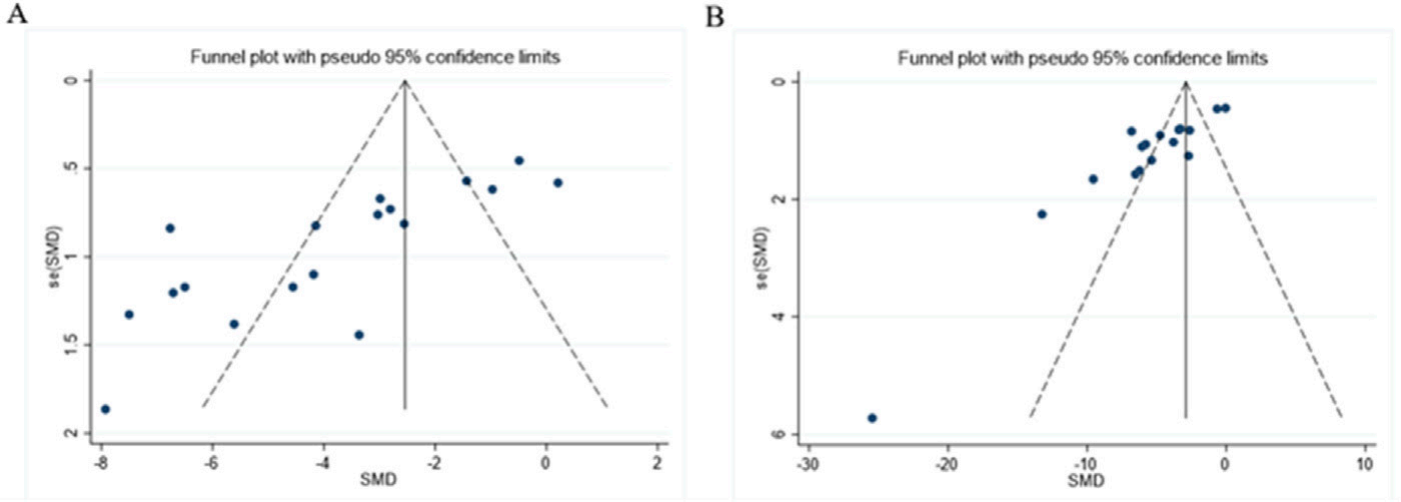
Funnel plot: effect of Rg1 on **(A)** ALT and **(B)** AST.

**FIGURE 13:**
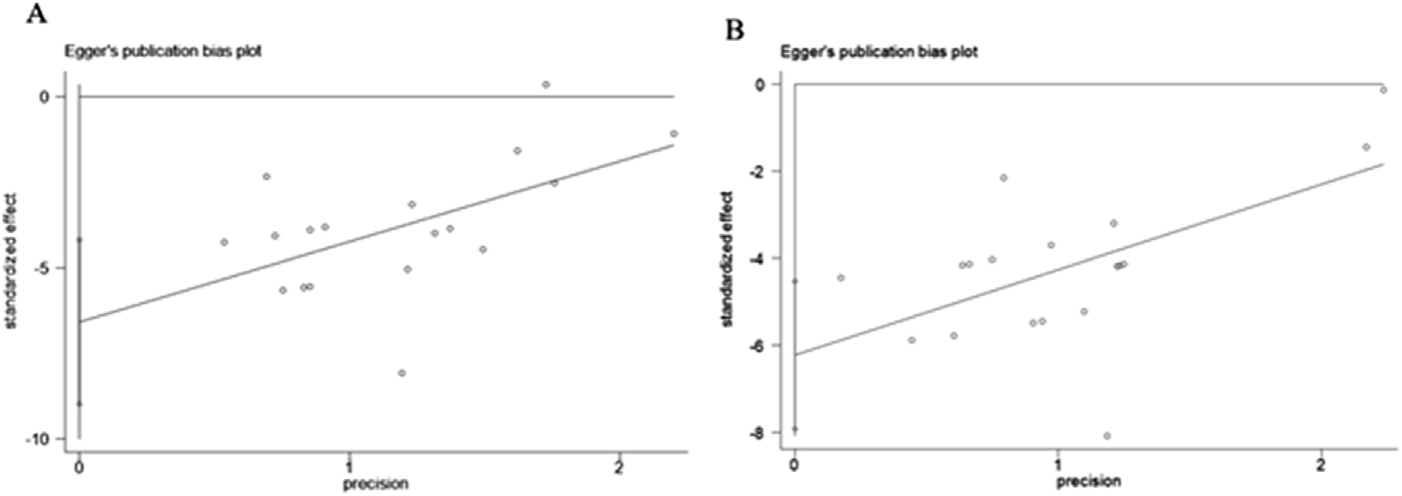
Egger’s publication bias plot for **(A)** ALT, **(B)** AST.

As presented in Figures 12A, B, the funnel plot indicated asymmetry in the comparison of ALT and AST levels. The Egger’s test results shown in Figures 13A, B revealed significant publication bias for both ALT (*P* < 0.05) and AST (*P* < 0.05). This bias may be attributed to the non-reporting of negative results and the relatively low quality of the included literature.

### 3.9 Time-dose analysis

**FIGURE 14:**
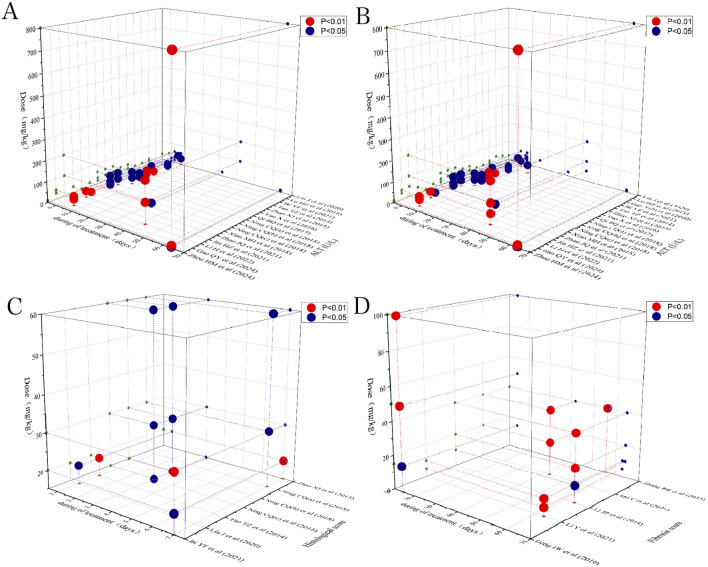
Scatter plot of the time-dose interval analysis on **(A)** ALT, **(B)** AST, **(C)** Histological, and **(D)** Fibrosis score.

To identify the most effective dose of preclinical Rg1 intervention for LI and LF, a time-dose analysis was performed, considering data on histological score, ALT, AST, and fibrosis score. The analysis indicated that treatment with Rg1 at doses ranging from 4 to 800 mg/kg/d for 1–64 days had a significant positive effect on ALT levels compared to the model group (*P* < 0.05). Similarly, Rg1 treatment at doses of 4–800 mg/kg/d for 1–64 days significantly reduced AST levels compared to the model group (*P* < 0.05). Further analysis showed that Rg1 treatment at doses of 20–60 mg/kg/d for 1–7 days had a better effect on histological score compared to the model group (*P* < 0.05). Additionally, treatment with Rg1 at doses ranging from 10 to 100 mg/kg/d for 14–63 days significantly improved the fibrosis score compared to the model group (*P* < 0.05). The results of the time-dose interval analysis suggested that the effective dose range for ginsenoside Rg1 is between 4 and 800 mg/kg/d, with treatment durations spanning 1–64 days (Figure 14).

## 4 Discussion

### 4.1 Effectiveness and summary of evidence

Traditional Chinese medicine (TCM) has a long history and is highly regarded for its mild and far-reaching curative effects, along with fewer side effects, making it a promising approach for disease management (Gan et al., 2023). Despite a large number of preclinical studies demonstrating that Rg1 exhibits a variety of biological activities, such as anti-inflammatory, anti-apoptotic, and antioxidant effects, as well as hepatoprotective action including mitigating the progression of nonalcoholic fatty liver disease, inhibiting viral hepatitis, countering LF, ameliorating LI from diverse etiologies, and reducing hepatocellular carcinoma, its reliability as a therapeutic agent for LI progressing to LF.

Remains inconsistent and insufficiently supported by evidence. This systematic review and meta-analysis integrates, for the first time, the preclinical evidence for the use of Rg1 in the treatment of LI and LF, confirming its potential therapeutic role in these conditions through a comprehensive meta-analysis. In this review, 24 preclinical studies involving a total of 423 animals were assessing with the primary objective of evaluating the therapeutic effects of Rg1 on LI and LF and elucidating the specific mechanisms by which LI progresses to LF in animal models. The overall methodological quality of the included studies was moderate. By summarizing and analyzing the various indicators, our meta-analysis found that Rg1 improved liver function indicators, such as ALT and AST; inflammation markers, such as TNF-α, IL-6, and IL-1β; apoptosis indicators, including BAX and Bcl-2; and oxidative stress indicators, such as SOD, MDA, GSH, Keap1, Nrf2, GCLM, GCLC, NQO1. However, significant heterogeneity was observed in the primary outcome indicators, including histological score, ALT, AST and LF score. According to the subgroup analysis, the heterogeneity may be attributed to differences in drug dosage, modeling method, duration of treatment, year of publication, and mode of administration.

### 4.2 Mechanism of action of Rg1 in the treatment of LI progressing to LF

**FIGURE 15:**
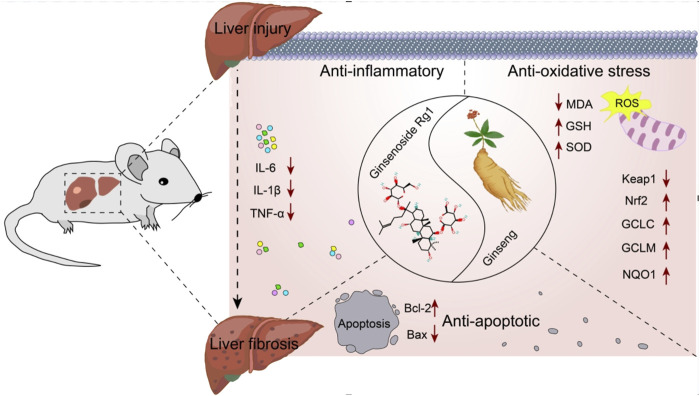
Possible mechanism of Rg1 on LI to LF.

Elucidating the molecular mechanisms underlying the role of Rg1 in the treatment of LI and LF is essential for advancing its clinical application. Therefore, the potential molecular mechanisms of Rg1 were comprehensively reviewed. Rg1 exerts hepatoprotective effects by inhibiting the Toll-like receptor 4 (TLR4) and NOD-like receptor thermal protein domain associated protein 3 (NLRP3)/nuclear factor kappa-B (NF-κB) signaling pathways. It also increases Nrf2 expression and translocation, enhances Bcl-2, and decreases BAX. These mechanisms are primarily reflected in Rg1’s anti-inflammatory, antioxidative stress, and anti-apoptotic properties (Figure 15).

#### 4.2.1 Anti-inflammatory effect

LI promotes inflammation and LF, with the initial stages involving hepatocyte damage, followed by the activation of paracrine secretion of inflammatory cells, and the autocrine activation of HSCs (Machado and Diehl, 2016). The progression from LI to LF is a complex pathophysiological process where inflammation and oxidative stress persist, playing critical roles (Feng et al., 2024; Sharma et al., 2024). Inflammation is closely associated with both acute and chronic liver diseases, acting in a dual capacity in the liver: it is essential for maintaining the health of the organism, but when uncontrolled, it becomes a major driver of liver pathology (Li et al., 2016). External or internal injury can trigger an inflammatory response, stimulating immune cells, which invade the liver and release various factors, advancing inflammation (Hu et al., 2016). In liver diseases, signaling pathways such as TLR4 and NF-κB activate endogenous cellular inflammatory vesicles, releasing pro-inflammatory cytokines like IL-1β, IL-6, and TNF-α. These cytokines promote a shift toward a type 2 inflammatory response, which, as liver disease progress, leads to tissue repair and the formation of LF (Taru et al., 2024). HSCs are the principal cells responsible for the production of extracellular matrix components, and their activation is central to both the progression and potential reversal of LF. Inflammation triggers the activation of HSCs through the secretion of pro-fibrotic factors like TGF-β1, which further amplifies LF in a positive feedback loop (Kisseleva and Brenner, 2021; Wang et al., 2024). Our results suggest that Rg1 inhibits the expression of TLR4 and downregulates NLRP3 inflammasome activity, along with pro-inflammatory cytokines TNF-α, IL-1β and IL-6 (Jin et al., 2021; Li et al., 2021; Ning et al., 2018c; Xin et al., 2016; Zhou et al., 2024).

#### 4.2.2 Anti-oxidative stress effect

Oxidative stress is a key factor in both LI and the development of LF (Crosas-Molist and Fabregat, 2015). It enhance the expression of collagen fibers and fibroblastogenic cytokines by activating HSCs. Activated HSCs produce large amounts of reactive oxygen species (ROS), and excessive ROS accumulation can trigger hepatocellular death, worsening inflammatory responses. This, in turn, activates NF-κB, which controls the transcription of pro-inflammatory cytokine genes, further aggravating liver inflammation and injury (Allameh et al., 2023; Kitsugi et al., 2023; Louvet and Mathurin, 2015). Nrf2, a key antioxidant transcription factor, plays a significant role in inflammation and chronic liver disease. During cellular oxidative stress, Nrf2 is activated and translocates to the nucleus, where it enhances cellular defense mechanisms by activating the expression of antioxidant genes, thereby mitigating oxidative damage (Chen et al., 2024). The Keap1/Nrf2 pathway is the most crucial antioxidant pathway. Under normal conditions, Nrf2 forms a stable dimer with its molecular chaperone Keap1 in the cytoplasm, remaining in a non-activated state. However, during oxidative stress, Nrf2 is activated, dissociates from Keap1 and translocates to the nucleus. There, it binds to antioxidant response elements and triggers the expression of key antioxidant genes, including NQO1, GCLM, GCLC, GSH, and SOD. This activation counteracts ROS-induced inflammation and oxidative stress, thereby safeguarding hepatocytes from further damage and preventing progression to LF (Chen et al., 2024). MDA, a major product of lipid peroxidation, is an indicator of increased lipid peroxidation in the liver (Gong et al., 2023). Our findings show that Rg1 significantly decreased MDA levels and increased the expression of SOD, GSH and Nrf2-regulated antioxidant genes like GCLC, GCLM and NQO1 (Gao et al., 2017a; Li et al., 2014; Ning et al., 2018a; Wei et al., 2018; Xiao et al., 2018).

#### 4.2.3 Anti-apoptosis effect

LI represents a complex pathological process frequently characterized by extensive hepatocyte apoptosis, which drives the progression to LF (Takehara et al., 2004; Brenner et al., 2013). Mitochondria, as central executors of apoptosis, primarily regulate this process through the Bcl-2 family of proteins, including Bcl-2 and BAX, which govern the permeability of the outer mitochondrial membrane (Hsu and Youle, 1997). Bcl-2 functions as an anti-apoptotic protein by inhibiting the release of apoptotic factors from mitochondria, whereas BAX overexpression accelerates apoptosis relative to Bcl-2 (Novo et al., 2006; Li et al., 2021). Therefore, modulation of Bcl-2/BAX signaling can effectively block hepatocyte apoptosis, thereby mitigating the transition from LI to LF. Rg1 was found to suppress BAX expression while enhancing Bcl-2 levels (Bi et al., 2021).

### 4.3 Limitations

Several limitations should be considered before interpreting the results of this study. First, the inclusion of only three high-quality English databases may introduce language bias; future studies should consider incorporating databases in multiple languages to mitigate this potential bias. Additionally, the quality of the animal studies included was generally moderate, with scores ranging from 5 to 7. Several studies mentioned randomization but did not provide details on blinding or allocation concealment. Third, significant heterogeneity was observed in some studies, and subgroup analysis suggested that variations in dosage, administration timing, mode of administration, and modeling methods may contribute to this.Therefore, larger preclinical studies are recommended to address these currently inconclusive aspects. Fourth, the complex pathogenic mechanisms underlying both LI and LF were only partially explored in this study, focusing primarily on the key mechanisms of Rg1 in treating these conditions. Moreover, some studies (Supplementary Table S4) did not report whether they underwent ethical review, complicating the assessment of the appropriateness of their animal experiment protocols. Finally, the evidence supporting Rg1 for treating LF is less robust than for LI, with only six animal studies reporting on Rg1’s effects on LF. Despite these limitations, the findings still suggest that Rg1 plays a beneficial role in the progression of LI to LF and holds promise as a therapeutic agent for both conditions.

## 5 Conclusion

Rg1 plays a beneficial role in the dynamic progression from LI to LF, exerting hepatoprotective effects through inhibition of the TLR4 and NLRP3/NF-κB signaling pathways, upregulation and translocation of Nrf2, as well as enhancing Bcl-2 expression and decreasing BAX levels. This study provides strong evidence supporting the beneficial effects of Rg1, which is well-documented for significantly reducing pathological scores, improving liver function, lowering pro-inflammatory markers, inhibiting oxidative stress, and preventing apoptosis in rodent models of LI and LF. Thus, Rg1 emerges as a promising drug candidate for treating both LI and LF, offering an evidence-based foundation for its potential development and clinical application. However, due to the observed heterogeneity and publication bias in the included studies, the results should be interpreted with caution. Further high-quality preclinical and clinical studies are essential to fully evaluate the therapeutic efficacy of Rg1.

## Glossary

LI: liver injury
LF: liver fibrosis
HSCs: hepatic stellate cells
ECM: extracellular matrix
Rg1: ginsenoside Rg1
Keap1: kelch-like ECH-associated protein 1
Nrf2: nuclear factor erythroid 2-related factor 2
HO-1: heme oxygenase-1
ALT: alanine aminotransferase
AST: aminotransferase
MDA: malondialdehyde
SOD: superoxide dismutase
GSH: glutathione
Nrf2: nuclear factor erythroid 2-related factor 2
GCLM: glutamate-cysteine ligase modifier subunit
GCLC: glutamate-cysteine ligase catalytic subunit
NQO1: NADH quinone xidoreductase 1
TNF-α: tumor necrosis factor-α
IL-6: interleukin 6
IL-1β: interleukin-1β
Bcl-2: B-cell lymphoma-2
BAX: BCL2-Associated X
HYP: hydroxyproline
α-SMA: α-smooth muscle actin
PCIII: procollagen typeIII
SMDs: standardized mean differences
95% CI: 95% confidence intervals
CCL4: carbon tetrachloride
TAA: thioacetamide
ANIT: naphthylisothiocyanate
TCDD: 2,3,7,8-tetrachlorodibenzo-p-dioxin
D-gal: d-galactose
APAP: acetaminophen
TCM: Traditional Chinese medicine
TLR4: Toll-like receptor 4
NLRP3: NOD-like receptor thermal protein domain associated protein 3; NF-κB;
ROS: reactive oxygen species.
