# Supplementary material for: Protective role of ginsenoside Rg1 in the dynamic progression of liver injury to fibrosis: a preclinical meta-analysis
Source: Front Pharmacol. 2025 Jan 28;16:1512184. doi: 10.3389/fphar.2025.1512184 (PMC11810943; doi:10.3389/fphar.2025.1512184)
Supplement: Supplementary file 1 [file Table1.docx]

**Supplementary materials**

**Supplementary Table 1**. Literature search strategy for Rg1 in the treatment of LI and LF

| **No. search items** |
| --- |
| 1. Liver Cirrhosis [Mesh] |
| 1. Liver injury [Mesh] |
| 1. Liver Cirrhosis [Title/Abtract] |
| 1. Hepatic Cirrhosis [Title/Abtract] |
| 1. Hepatic Fibrosis [Title/Abtract] |
| 1. Cirrhosis [Title/Abtract] |
| 1. Cirrhosis Hepatic [Title/Abtract] |
| 1. Hepatocirrhosis [Title/Abtract] |
| 1. Liver Damage [Title/Abtract] |
| 1. Hepatotoxicity [Title/Abtract] |
| 1. Hepatic Injury [Title/Abtract] |
| 1. 1 OR 2 OR 3 0R 4 OR 5 OR 6 OR 7 OR 8 OR 9 OR 10 OR 11 |
| 1. Ginsenoside Rg1 [Mesh] |
| 1. Rg1 [Title/Abtract] |
| 1. 13 OR 14 |
| 1. 12 AND 15 |

**Supplementary Table 2.** Methodological quality of included animal studies.

| **Study ID** | **A** | **B** | **C** | **D** | **E** | **F** | **G** | **H** | **I** | **J** | **K** |
| --- | --- | --- | --- | --- | --- | --- | --- | --- | --- | --- | --- |
| Zhang RR et al (2023) | 0 | 1 | 0 | 0 | 0 | 1 | 0 | 1 | 1 | 1 | 5 |
| Li Y et al (2021) | 1 | 1 | 0 | 1 | 0 | 1 | 0 | 1 | 1 | 1 | 7 |
| Mo C et al (2021) | 1 | 1 | 0 | 0 | 0 | 1 | 0 | 1 | 1 | 1 | 6 |
| Wei XY et al (2018) | 1 | 1 | 0 | 1 | 0 | 1 | 0 | 1 | 1 | 1 | 7 |
| Li JP et al (2014) | 1 | 1 | 0 | 1 | 0 | 1 | 0 | 1 | 1 | 1 | 7 |
| Geng JW et al (2010) | 0 | 1 | 0 | 1 | 0 | 1 | 0 | 1 | 1 | 1 | 6 |
| Zhou HM et al (2024) | 1 | 1 | 0 | 1 | 0 | 1 | 0 | 1 | 1 | 1 | 7 |
| Gao QY et al (2024) | 0 | 1 | 0 | 1 | 0 | 1 | 0 | 1 | 1 | 1 | 6 |
| Li H et al (2022) | 1 | 1 | 0 | 1 | 0 | 1 | 0 | 1 | 1 | 1 | 7 |
| Jin HZ et al (2021) | 1 | 1 | 0 | 1 | 0 | 1 | 0 | 1 | 1 | 1 | 7 |
| Zhao JQ et al (2021) | 0 | 1 | 0 | 1 | 0 | 1 | 0 | 1 | 1 | 1 | 6 |
| Xiao MH et al (2018) | 1 | 1 | 0 | 1 | 0 | 1 | 0 | 1 | 1 | 1 | 7 |
| Ning CQ et al (c) (2018) | 1 | 1 | 0 | 1 | 0 | 1 | 0 | 1 | 1 | 1 | 7 |
| Ning CQ et al (b) (2018) | 1 | 1 | 0 | 1 | 0 | 1 | 0 | 1 | 1 | 1 | 7 |
| Ning CQ et al (a) (2018) | 0 | 1 | 0 | 1 | 0 | 1 | 0 | 1 | 1 | 1 | 6 |
| Qi BQ et al (2017) | 1 | 1 | 0 | 1 | 0 | 1 | 0 | 1 | 1 | 1 | 7 |
| Xin Y et al (2016) | 1 | 1 | 0 | 1 | 0 | 1 | 0 | 1 | 1 | 1 | 7 |
| Zhao XJ et al (2015) | 0 | 1 | 0 | 1 | 0 | 1 | 0 | 1 | 1 | 1 | 6 |
| Tao TZ et al (2014) | 1 | 1 | 0 | 1 | 0 | 1 | 0 | 1 | 1 | 1 | 7 |
| Bi YF et al (2021) | 1 | 1 | 0 | 1 | 0 | 1 | 0 | 1 | 1 | 1 | 7 |
| Gao Y et al (a) (2020) | 0 | 1 | 0 | 1 | 0 | 1 | 0 | 1 | 1 | 1 | 6 |
| Gao Y et al (b)2020 | 1 | 1 | 0 | 1 | 0 | 1 | 0 | 1 | 1 | 1 | 7 |
| Lu HZ et al (2018) | 1 | 1 | 0 | 1 | 0 | 1 | 0 | 1 | 1 | 1 | 7 |
| Lin J et al (2020) | 1 | 1 | 0 | 1 | 0 | 1 | 0 | 1 | 1 | 1 | 7 |

A. sequence generation; B. baseline characteristic; C. allocation concealment; D. random housing; E. blinding investigators; F. random outcome assessment; G. blinding outcome assessor; H. incomplete outcome data; I. selective outcome reporting; J. other sources of bias; K. total.

**Supplementary Table 3.** The subgroup analysis of histological score, ALT, AST, and Fibrosis score.

| **Indicators** | **Subgroup** |  | **No. studies** | **SMD [95% CI]** | ***I*^2^** | ***P*** |
| --- | --- | --- | --- | --- | --- | --- |
| ALT | ALT | before 2019 | 11 | -3.04 [-4.33, -1.75] | 88% | <0.01 |
|  |  | after 2019 | 7 | -4.21 [-5.39, -3.02] | 58% | <0.01 |
|  | Animal species | rat | 1 | -2.65 [-4.10, -1.21] | / | / |
|  |  | mice | 17 | -3.56 [-4.68, -2.43] | 87% | <0.01 |
|  | Dosage | <40 mg | 8 | -3.18 [-4.46, -1.90] | 79% | <0.01 |
|  |  | ≥ 40 mg | 10 | -3.82 [-5.46, -2.16] | 90% | <0.01 |
|  | Modeling  Method | Toxic | 14 | -4.15 [-5.47,-2.84 ] | 86% | <0.01 |
|  |  | Nutritional | 1 | -0.47 [-1.36,0.42 ] | / | / |
|  |  | Surgical | 3 | -1.89 [-3.04,-0.75 ] | 49% | <0.01 |
|  | Duration of treatment | < 7 days | 8 | -3.81 [-5.78, -1.84] | 89% | <0.01 |
|  |  | ≥ 7 days | 10 | -3.17 [-4.41, -1.94] | 84% | <0.01 |
|  | Method of administration | Intragastric | 8 | -4.24 [-6.15, -2.32] | 88% | <0.01 |
|  |  | injection | 10 | -3.02 [-4.35, -1.68] | 86% | <0.01 |
| AST | Year | before 2019 | 11 | -4.13 [-5.73, -2.53] | 90% | <0.01 |
|  |  | after 2019 | 7 | -5.20 [-7.10, -3.31] | 75% | <0.01 |
|  | Animal species | rat | 1 | -3.13 [-4.72, -1.54] | / | / |
|  |  | mice | 17 | -4.72 [-6.16, -3.28] | 90% | <0.01 |
|  | Dosage | <40 mg | 8 | -4.01 [-5.46, -2.55] | 76% | <0.01 |
|  |  | ≥ 40 mg | 10 | -4.98 [-7.00, -2.97] | 92% | <0.01 |
|  | Modeling  Method | Toxic | 14 | -5.47 [-7.26, -3.68] | 90% | <0.01 |
|  |  | Nutritional | 1 | -0.64 [-1.54, 0.27 ] | / | / |
|  |  | Surgical | 3 | -2.96 [-3.96, -1.97 ] | 0% | <0.01 |
|  | Duration of treatment | < 7 days | 8 | -4.40 [-6.67, -2.14] | 92% | <0.01 |
|  |  | ≥ 7 days | 10 | -4.79 [-6.57, -3.01] | 89% | <0.01 |
|  | Method of administration | Intragastric | 8 | -5.60 [-8.11, -3.08] | 89% | <0.01 |
|  |  | injection | 10 | -4.06 [-5.79, -2.33] | 90% | <0.01 |
| Histological score | Year | before 2019 | 5 | -8.18 [-10.96, -5.40] | 62% | <0.01 |
|  |  | after 2019 | 2 | -4.32 [-7.54, -1.11] | 69% | <0.01 |
|  | Animal species | rat | 1 | -2.98 [-4.52, -1.44] | / | / |
|  |  | mice | 6 | -7.74 [-9.97, -5.51] | 54% | <0.01 |
|  | Dosage | <40 mg | 4 | -4.82 [-7.05, -2.60] | 57% | <0.01 |
|  |  | ≥ 40 mg | 3 | -9.18[-11.48, -6.89] | 14% | <0.01 |
|  | Modeling Method | Toxic | 4 | -8.41 [-10.56, -6.25 ] | 31% | <0.01 |
|  |  | Surgical | 3 | -4.37 [-6.93, -1.80 ] | 58% | <0.01 |
|  | Duration of treatment | < 7 days | 4 | -6.65 [-10.22, -3.09] | 86% | <0.01 |
|  |  | ≥ 7 days | 3 | -7.28 [-9.67, -4.89] | 0% | <0.01 |
|  | Method of administration | Intragastric | 3 | -7.56 [-9.51, -5.61] | 0% | <0.01 |
|  |  | injection | 4 | -6.98 [-9.49, -4.47] | 77% | <0.01 |
| Fibrosis score | Year | before 2019 | 2 | -2.65 [-3.55, -1.75] | 0% | <0.01 |
|  |  | after 2019 | 3 | -5.26 [-8.21, -2.30] | 63% | <0.01 |
|  | Animal species | rat | 2 | -2.65 [-3.55, -1.75] | 0 | <0.01 |
|  |  | mice | 3 | -5.26 [-8.21, -2.30] | 63% | <0.01 |
|  | Dosage | <40 mg | 1 | -3.74 [-6.66, -0.82] | / | / |
|  |  | ≥ 40 mg | 4 | -3.70 [-5.41, -2.00] | 70% | <0.01 |

**Supplementary Table 4.** Ethical information and approval number for each study

| **Study (Years)** | **Ethics adoption unit** | **Approval Number** |
| --- | --- | --- |
| Zhang RR et al (2023) | the University Animal Care and Use Committee. | Unkown |
| Li Y et al (2021) | the Animal Ethics Committee of Anhui Medical University. | LLSC20160183 |
| Mo C et al (2021) | the Animal Care and Ethics Committee Southern Medical University. | Unkown |
| Wei XY et al (2018) | the Ethics Committee for Animal Experiments of Chongqing Medical University. | Unkown |
| Li JP et al (2014) | the Animal Care Committee of the Peking Union Medical College. | Unkown |
| Geng JW et al (2010) | the Animal Care and Use Committee of Yunnan University. | Unkown |
| Zhou HM et al (2024) | the Laboratory Animal Ethics Committee of Anhui Medical University. | LLSC20232095 |
| Gao QY et al (2024) | the Animal Ethics Committee of Tongji Hospital Affiliated Tongji Medical College. | TJH-202106008 |
| Li H et al (2022) | the Animal Ethics Committee of China Academy of Chinese Medical Sciences. | 2021B309 |
| Jin HZ et al (2021) | the Committee of Experimental Animals of Wenzhou People's Hospital. | XXGK20190724 |
| Zhao JQ et al (2021) | the institutional ethic committee. | Unkown |
| Xiao MH et al (2018) | the Chongqing Medical University Animal Care and Use Committee. | Unkown |
| Ning CQ et al (c) (2018) | the Institutional Animal Care and Use Committee at Dalian Medical University. | Unkown |
| Ning CQ et al (b) (2018) | the Ethical Committee and the China National Institutes of Healthy Guidelines. | Unkown |
| Ning CQ et al (a) (2018) | the Ethical Committee and the China National Institutes of Healthy Guidelines. | Unkown |
| Qi BQ et al (2017) | the animal ethics committee of Bengbu Medical College. | Unkown |
| Xin Y et al (2016) | the Animal Care and Use Committee of China Pharmaceutical University. | Unkown |
| Zhao XJ et al (2015) | the Ethical Committee of University of Macau. | Unkown |
| Tao TZ et al (2014) | the Institutional Animal Care and Use Committee of Changhai Hospital. | CH 20120815-08 |
| Bi YF et al (2021) | Unkown | Unkown |
| Gao Y et al (a) (2020) | the Animal Care Committee of the Peking Union Medical College. | Unkown |
| Gao Y et al (b)2020 | Unkown | Unkown |
| Lu HZ et al (2018) | the animal ethics committee of Wuhan Third Hospital. | KY2018—026 |
| Lin J et al (2020) | the Animal Ethics Committee of Kunming Medical University. | Unkown |
